# Systematic Review of Percutaneous and Transcutaneous Posterior Tibial Neurostimulation for Lower Urinary Tract Symptoms & Lower Urinary Tract Dysfunction in Children

**DOI:** 10.1002/nau.70264

**Published:** 2026-03-15

**Authors:** Zoe S. Gan, Suhaib Abdulfattah, Jennifer Lege‐Matsuura, Katherine M. Fischer, Jason P. Van Batavia

**Affiliations:** ^1^ Department of Surgery, Division of Urology University of Pennsylvania Perelman School of Medicine Philadelphia Pennsylvania USA; ^2^ Department of Urology Stanford University School of Medicine Palo Alto California USA; ^3^ Department of Surgery, Division of Urology The Children's Hospital of Philadelphia Philadelphia Pennsylvania USA; ^4^ Holman Biotech Commons University of Pennsylvania Libraries Philadelphia Pennsylvania USA

**Keywords:** lower urinary tract dysfunction, lower urinary tract symptoms, neuromodulation, pediatrics, tibial nerve stimulation

## Abstract

**Objective:**

To systematically evaluate the efficacy of posterior tibial nerve stimulation (TNS) in children with lower urinary tract symptoms (LUTS) and/or lower urinary tract dysfunction (LUTD).

**Materials and Methods:**

A systematic review was conducted following PRISMA guidelines. Databases including PubMed, EMBASE, CENTRAL, and Scopus were searched for relevant studies published before May 11, 2023. Studies included pediatric patients (< 18 years old) treated with percutaneous or transcutaneous posterior TNS (PTNS or TTNS, respectively) for LUTS or LUTD. Study quality was assessed using the Mixed Methods Appraisal Tool (MMAT), and data were synthesized qualitatively. Studies were categorized as mixed neurogenic (NLUTD) and non‐neurogenic (NN LUTD), monosymptomatic nocturnal enuresis (MNE), and NN LUTD.

**Results:**

Of 576 studies, 26 met the inclusion criteria. For mixed NLUTD/NN LUTD, PTNS had variable efficacy (0%−100%) for improving LUTS, and TTNS had at least some urinary symptom improvement for around 80% of patients. For MNE, most studies showed a mild improvement of 1−2 wet nights over 1−2 weeks with TNS. For NN LUTD, TNS led to improvement in LUTS in around 40%−70% of patients, although complete cure rates were variable. Adverse events were minimal, with only transient discomfort reported. The overall quality of evidence was low to moderate, and most studies had small sample sizes and lacked comparison groups. No studies directly compared PTNS and TTNS.

**Conclusions:**

Overall, posterior TNS via either a percutaneous or transcutaneous approach often provides at least a mild benefit to pediatric patients with LUTS and LUTD. However, the variability in study outcomes and low quality of evidence suggest a need for more robust studies with standardized outcomes and comparison groups to better establish the efficacy and role of TNS in children.

## 1 Introduction

Lower urinary tract symptoms (LUTS) and lower urinary tract dysfunction (LUTD) are common in pediatric populations and can significantly impact the quality of life (QOL) of both children/adolescents and their families [1, 2, 3]. LUTS refer to abnormal bladder or urinary symptoms, such as incontinence, urgency, or increased frequency, and can be classified according to storage and/or voiding phases [4]. While LUTS can occur once or variably, the term “LUTD,” or bladder dysfunction, refers to a broader functional abnormality in the lower urinary tract when LUTS are clinically significant. LUTD can be caused by many conditions, which may be divided into non‐neurogenic (NN LUTD) and neurogenic (NLUTD) based on the underlying etiology [4]. The vast majority of LUTD, especially when non‐neurogenic in origin, is managed conservatively with behavioral interventions (standard urotherapy) [5, 6] or with pharmacological interventions [7].

For refractory LUTD, an alternative treatment is neuromodulation, which until recently was considered third‐line therapy in adults with LUTD [8] and rarely used in pediatric patients. Neuromodulation involves stimulating specific nerves to alter neurological pathways involved in the micturition cycle and normalize bladder function [9]. This may be performed noninvasively via transcutaneous stimulation, minimally invasively via percutaneous stimulation, or in an invasive manner via a surgically implanted neurostimulator. While different nerves can be stimulated, most studies on peripheral neuromodulation for LUTD involve stimulation of the posterior tibial nerve. In 1983, Maguire et al. first described posterior tibial nerve stimulation (TNS) via transcutaneous approach (TTNS) [10]. Stoller, in 1999, was the first to describe a percutaneous technique for posterior tibial nerve stimulation (PTNS) [11]. Although much is still unknown about the exact mechanism of action, TNS is thought to modulate the sacral nerve reflexes [12] and offers a less invasive alternative to implantable sacral nerve stimulators.

While TNS has demonstrated improvements in LUTS and QOL in adults, particularly in overactive bladder (OAB) [13] and NLUTD [14], there is limited data on its utility in pediatric patients for different types of LUTD. Given the need to establish evidence for TNS in pediatrics to inform future research and pediatric‐specific guidelines, our study aims to systematically evaluate the efficacy of TNS in children with LUTS or LUTD. We hypothesized that TNS would lead to subjective and objective improvement in the majority of pediatric patients treated.

## 2 Materials & Methods

### 2.1 Search Strategy

The systematic review was performed according to the Preferred Reporting Items for Systematic Reviews and Meta‐Analyses (PRISMA) guidelines. A medical research librarian searched PubMed, EMBASE, CENTRAL, and Scopus for studies published between database inception and May 11, 2023. Searches combined controlled vocabulary (MESH, EMTREE) with keywords. We identified studies from these databases that qualified for all three key concepts (TNS, children, and LUTD). Each concept was built on a list of related search terms for medical subject headings, title, and abstract to maximize the sensitivity of the search (Supporting Information Material S1). The grey literature was also searched for unpublished abstracts from related specialty society meetings (American Urological Association, International Continence Society, Society for Pediatric Urology, and International Children's Continence Society).

### 2.2 Eligibility Criteria

Studies were included if they were performed in a pediatric population (age < 18 years), involved percutaneous or transcutaneous stimulation of the tibial nerve, and designated LUTS or LUTD as the indication for treatment. Studies were excluded if they were not written in English, did not report original data, included an adult population, reported on parasacral transcutaneous electrical nerve stimulation (PTENS) or a surgically implanted neurostimulator without comparison to TNS, or if the indication for treatment was not urologic (e.g., fecal incontinence).

### 2.3 Study Screening and Data Extraction

Covidence systematic review software was used to screen references, first by title and abstract, then by full text review. Studies were screened independently by two authors (Z.S.G. and K.M.F.) with a third author (J.P.V.B.) as a tiebreaker. Data extraction was performed independently by two authors (Z.S.G. and S.A.F.) with a third author (J.P.V.B.) as a tiebreaker. Data were extracted on study design, publication type, study population (including demographics, indication for treatment, and prior treatments), treatment details, and reported outcomes.

### 2.4 Quality Assessment

The overall quality of studies and risk of bias were assessed using the Mixed Methods Appraisal Tool (MMAT) [15], which was chosen to accommodate the variety of methods used in the included studies. Two authors (Z.S.G. and S.A.F.) independently rated study quality domains with a third author (J.P.V.B.) as a tiebreaker. As suggested by the creators of the MMAT, an overall score of 1−5 was given to each study based on the number of quality criteria met, and ratings for each criterion are reported in detail [16]. As no guidance for qualitative interpretation of these scores has been offered, we interpreted overall study quality as high (score 5), medium (scores 3−4), or low (scores 1−2). Given differences in outcome measures and reporting of summary statistics among studies, heterogeneity was assessed qualitatively [17]. The Grading of Recommendations, Assessment, Development, and Evaluations (GRADE) approach was used to assess the certainty of evidence for outcomes of interest with adequate data.

### 2.5 Data Synthesis

Data were summarized qualitatively. The studies were grouped based on indication: mixed NLUTD/NN LUTD, monosymptomatic nocturnal enuresis (MNE), and NN LUTD. For “mixed NLUTD and NN LUTD,” studies including NLUTD were grouped with studies including both NLUTD and NN LUTD due to (1) a low number of NLUTD patients in general, (2) the inability to separate neurogenic from non‐neurogenic patients in all studies, and (3) the ability to compare the effect of TNS between neurogenic and non‐neurogenic patients more effectively within studies.

## 3 Results

**Figure 1:**
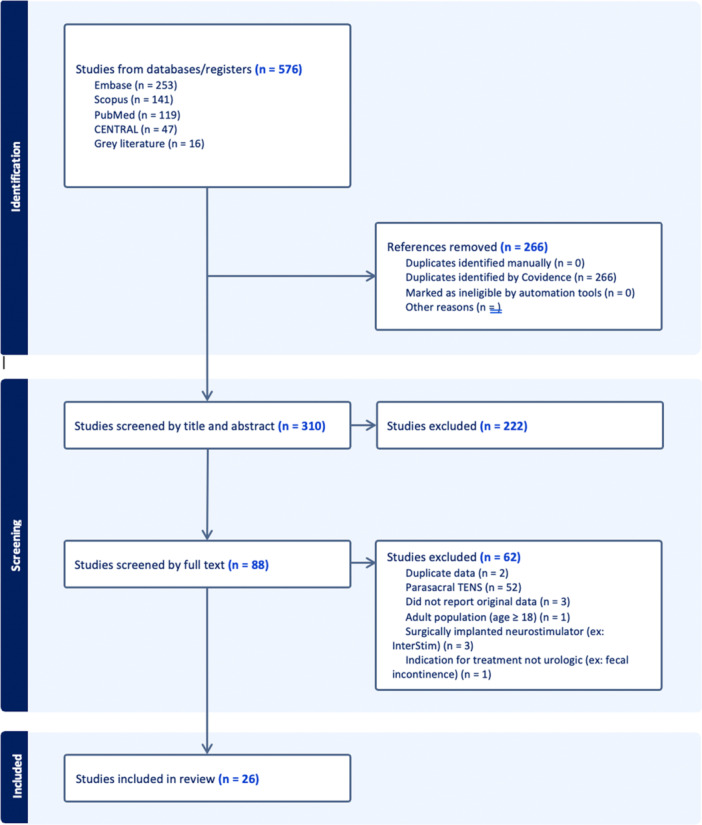
PRISMA flow diagram showing the study selection process. PRISMA, Preferred Reporting Items for Systematic Reviews and Meta‐Analyses; TENS, transcutaneous electrical nerve stimulation.

**Figure 2:**
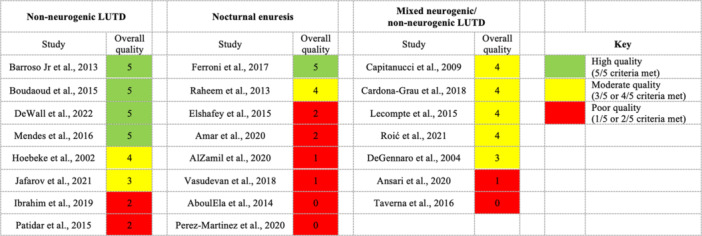
Summarized Mixed Methods Assessment Tool quality assessment ratings for included studies. LUTD, lower urinary tract dysfunction.

Of 576 studies identified by the search strategy, 26 were ultimately included in the review. The systematic screening process is depicted in the PRISMA diagram (Figure 1). Quality assessment data is summarized in Figure 2 and detailed in Supporting Information S1: Table 1.

### 3.1 Population Characteristics

Seven studies included children with NLUTD (five of which also included NN LUTD), encompassing 78 patients with NLUTD, 76 patients with NN LUTD, and 28 patients unspecified. Patient characteristics are reported in Table 1. Etiologies of NLUTD included spina bifida [18, 19], spina bifida occulta [20, 21], sacral agenesis [22], and other pathologies including spinal cord or pelvic tumor, tethered cord syndrome, spinal cord injury, and transverse myelitis [18], and unspecified [22, 23]. Nine studies included children with MNE, encompassing 311 patients (Table 2), and eight studies included exclusively children with non‐neurogenic LUTD, encompassing 326 patients (Table 3).

**Table 1:** Characteristics of studies on tibial nerve stimulation in mixed neurogenic/non‐neurogenic LUTD cohorts.

Study	*N*	Study type	Age (years)	Female sex	Indication	Prior treatments	Prior AC (yes/no)	Current AC (yes/no)	Treatment type	Comparison group	Treatment details	Follow‐up	Quality Score^a^
Capitanucci et al. 2009	55 NN LUTD: 43 NLUTD: 12	P	NLUTD: mean 13.5, SD 9.02 NN LUTD: mean 10.7, SD 4.8	NLUTD: 33% NN LUTD: 58%	NLUTD NN LUTD	12 months of conventional treatment (AC or alpha blockers, behavioral techniques, bladder training, pelvic floor relaxation exercises +/‐biofeedback and chemoprophylaxis NLUTD: 4/12 on CIC	NR	NR	PTNS	None	30 min weekly for 12 weeks	12 weeks 1 year 2 years	4
Cardona‐Grau et al. 2018	28	P	Mean 10.7, range 7−18	61%	NLUTD NN LUTD NE	Behavioral urotherapy	NR	NR	TTNS	None	20 min twice weekly for 12 weeks	12 weeks	4
Lecompte et al. 2015	8 (3 with UI)	P	Mean 11.75, range 10−13	37.5%	NLUTD All had FI in the setting of bowel malformation or neurologic pathology; 3/8 (37.5%) had UI	FI: At least 2 years of transit regulators, AC if needed, anterograde enemas, transanal irrigations, abdominal and perineal rehabilitation, and psychological support UI: 2 years of AC, 1 year on CIC with Mitrofanoff	Yes	No	TTNS	None	20 min daily planned for 6 months; actual mean 9 months (range 7−14), 10 Hz	2 months 6 months	4
Roić et al. 2021	39	P	Mean 13.03, SD 5.04, range 4−21	51%	NLUTD	NR	NR	NR	TTNS	None	30 min daily for 12 weeks	12 weeks	4
DeGennaro et al. 2004	23 OAB: 10 NN UR: 7 NLUTD: 6	C	OAB: mean 9.1, SD 2.4 NN UR: mean 12, SD 4 NLUTD: mean 9.2, SD 2.9	OAB: 40% NN UR: 47% NLUTD: 67%	NLUTD NN LUTD	OAB: At least 2 years of AC NN UR: kinesitherapy, micturition biofeedback, doxazosin, timed voiding, 1 (14%) on CIC NLUTD: 4 (67%) on CIC	Yes	NR	PTNS	None	30 min weekly for 12 weeks, 20 Hz/ 0−10 mA/200 µs	12 weeks	3
Ansari et al. 2020	21 OAB: 12 DU: 9	P	Mean 5.6, range 4−16	NR	UDS‐proven DO or DU	NR	NR	NR	PTNS	None	30 min weekly for 12 weeks	12 weeks	1
Taverna et al. 2016	8 NLUTD: 4 NN LUTD: 4	P	NLUTD: mean 11 NN LUTD: mean 9.5	NLUTD: 75% NN LUTD: 50%	NLUTD NN LUTD	Resistant to AC treatment	NR	NR	PTNS	None	30 min twice weekly for 12 weeks	12 weeks 15 weeks 24 weeks 9 months 3.25 years	0

Abbreviations: AC, anticholinergic medication; C, cohort study, unclear if prospective or retrospective; CIC, clean intermittent catheterization; DU, detrusor underactivity; FI, fecal incontinence; LUTD, lower urinary tract dysfunction; N, number of patients; NE, nocturnal enuresis; NLUTD, neurogenic lower urinary tract dysfunction; NN LUTD, non‐neurogenic lower urinary tract dysfunction; NR, not reported; P, prospective cohort study; SD, standard deviation; UI, urinary incontinence; UR, urinary retention.

^a^ Quality score based on the Mixed Methods Assessment Tool: high (5/5 criteria met); moderate (3/5 or 4/5 criteria met); poor (0−2/5 criteria met).

**Table 2:** Characteristics of studies on tibial nerve stimulation in nocturnal enuresis cohorts.

Study	*N*	Study type	Age (years)	Female sex	Indication	Prior treatments	Prior AC (yes/no)	Current AC (yes/no)	Treatment type	Comparison group	Treatment details	Follow‐up	Quality Score^a^
Ferroni et al. 2017	22	P	Mean 11.4, range 7−16	45.45%	NE	8 (36%) on desmopressin 1 (0.5%) on bedwetting alarm 5 (23%) on both desmopressin and bedwetting alarm 2 (9%) on oxybutynin	Yes	NR	TTNS	None	60 min daily for 2 weeks, 5 Hz/0−100 mA/200 µs	2 weeks pre‐stimulation 2 weeks during stimulation 2 weeks post‐stimulation	5
Raheem et al. 2013	28	R	PTNS: mean 13.7, SD 2.8 Sham: mean 14, SD 2.8	PTNS: 43% Sham: 36%	NE	6 months of conventional and combination therapy (desmopressin, anticholinergics, and alarm)	NR	NR	PTNS	Sham	PTNS: 30 min weekly for 12 weeks. Sham: 30 min weekly for 12 weeks	12 weeks 14 weeks	4
Elshafey et al. 2015	80 TTNS: 40 CG: 40	R	TTNS: mean 6.32, SD 0.65 CG: mean 6.5, SD 0.42	TTNS: 50% CG: 57.5%	NE	NR	NR	NR	TTNS	CG: Bedwetting alarm for 12 weeks	3x per week for 12 weeks, 20 Hz/0−10 mA/200 µs	12 weeks	2
Amar et al. 2020	80	C	NR	NR	NE	NR	NR	NR	PTNS	Desmopressin	Weekly for 12 weeks	12 weeks 4 months	2
Al Zamil et al. 2020	20	C	Range 10−13	NR	NE	NR	NR	NR	TTNS	None	High‐frequency: 100 Hz/100 µs Low‐frequency: 1 Hz/200 µs	NR	1
Vasudevan et al. 2018	53	R	Mean 10.2, SD 2.5	NR	NE	1 month of urotherapy (no further details)	NR	NR	TTNS	Sham, PTENS	20 min nightly for 30 days	30 days	1
Aboul Ela et al. 2014	20	C	NR	NR	NE	NR	NR	NR	PTNS	None	30 min weekly for 12 weeks, 20 Hz	12 weeks	0
Perez‐Martinez et al. 2020	8	R	PTNS: mean 10.5, SD 1.3 Sham: mean 10.7, SD 1.8	PTNS: 50% Sham: 50%	NE	6 months of first‐ or second‐line treatments (unspecified)	NR	NR	PTNS	Sham	PTNS: 30 min, 3x per week for 2 weeks, 20 Hz/1−9 mA/200 µs Sham: 30 min, 3x per week for 2 weeks	2 weeks	0

Abbreviations: AC, anticholinergic medication; C, cohort study, unclear if prospective or retrospective; CG, control group; N, number of patients; NE, nocturnal enuresis; NR, not reported; P, prospective cohort study; R, randomized control trial; SD, standard deviation.

^a^ Quality score based on the Mixed Methods Assessment Tool: high (5/5 criteria met); moderate (3/5 or 4/5 criteria met); poor (0−2/5 criteria met).

**Table 3:** Characteristics of studies on tibial nerve stimulation in non‐neurogenic LUTD cohorts.

Study	*N*	Study type	Age (years)	Female sex	Indication	Prior treatments	Prior AC (yes/no)	Current AC (yes/no)	Treatment type	Comparison group	Treatment details	Follow‐up	Quality Score^a^
Barroso Jr. et al. 2013	59 PTNS: 22 PTENS: 37	P	PTNS: mean 8.4, SD 3.8 PTENS: mean 7.5, SD 2.8	PTNS: 68% PTENS: 68%	OAB	No	No	No	PTNS	PTENS	PTNS: 30 min weekly for 12 weeks, 20 Hz/400 µs PTENS: 20 min, 3x per week for 7 weeks, 10 Hz/700 µs	12 weeks	5
Boudaoud et al. 2015	20 TTNS: 11 Sham: 9	R	TTNS: mean 11 Sham: mean 10	TTNS: 55% Sham: 45%	OAB	At least 6 months of anticholinergics	Yes	No	TTNS	Sham	TTNS: 2x weekly for 12 weeks, 10 Hz/10 mA/200 µs Sham: 2x weekly for 12 weeks	12 weeks Incontinence diary at 6 weeks	5
DeWall et al. 2022	101	M	Mean 9.7, SD 2.4	41%	NN LUTD	95% on urotherapy, 88% on antimuscarinics, 50% pelvic floor therapy	Yes	NR	PTNS	None	30 min weekly for 12 weeks, 20 Hz/0−10 mA/200 µs	12 weeks	5
Mendes et al. 2016	19	C	Mean 13.5, SD 2.7 years, range 7−17	74%	Chronic urinary retention	6 months of bladder regimen (unspecified), kinesitherapy, and biofeedback	NR	NR	PTNS	None	30 min weekly for 12 weeks	12 weeks	5
Hoebeke et al. 2002	32	P	Mean 11.7	47%	NN bladder sphincter dysfunction	At least 2 years of relaxation biofeedback, toilet posture correction, maintenance of a voiding and drinking schedule, uroflowmetry, and urine flow biofeedback Pharmacotherapy (antispasmodic & anticholinergics)	Yes	Yes “continued in 24 children (75%)”	PTNS	None	30 min weekly planned for 6−20 weeks, 20 Hz Mean = 10 weeks	Mean = 10 weeks	4
Jafarov et al. 2021	44	R	TTNS: median 8.5, IQR 5.7−8.7 Sham: median 8.4, IQR 6.8−11.5 CG: median 8.0, IQR 5.8−14.3	TTNS: 50% Sham: 50% CG: 7%	NN LUTD	Medical treatment for FVD “off for at least 3 months before treatment”	No	NR	TTNS	Sham CG: Healthy children without FVD or LUTS	TTNS: 30 min weekly for 12 weeks, 10−25 mA, gradually increased by 1 mA intervals Sham: 30 min weekly for 12 weeks, 0 mA	14 weeks 2 years	3
Ibrahim et al. 2019	20	P	Mean 7, SD 3.3	60%	OAB	NR	NR	NR	PTNS	None	30 min weekly for 12 weeks	12 weeks 6 months	2
Patidar et al. 2015	40	R	TTNS: mean 7.71, SD 2.22 Sham: mean 8.38, SD 2.27	60%	OAB	Behavioral therapy and at least 6 months of anticholinergics	Yes	Yes “continued in 24 children (60%)”	TTNS	Sham	TTNS: 30 min weekly for 12 weeks, 20 Hz/0−10 mA/200 µs Sham: 30 min weekly for 12 weeks	12 weeks	2

Abbreviations: AC, anticholinergic medication; C, cohort study, unclear if prospective or retrospective; CG, control group; CIC, clean intermittent catheterization; DO, detrusor overactivity; DU, detrusor underactivity; FVD, functional voiding disorder; IQR, interquartile range; LUTD, lower urinary tract dysfunction; M, mixed methods study; N, number of patients; NN, non‐neurogenic; NR, not reported; O, overactive bladder, OAB, overactive bladder; P, prospective cohort study; R, randomized control trial; SD, standard deviation; UDS, urodynamic studies; UI, urinary incontinence; UR, urinary retention.

^a^ Quality score based on the Mixed Methods Assessment Tool: high (5/5 criteria met); moderate (3/5 or 4/5 criteria met); poor (0−2/5 criteria met).

### 3.2 Intervention

TNS was delivered percutaneously in 50%−70% of studies in each group and transcutaneously in the remaining studies. Most treatments were administered for 20−30 min once or twice weekly for 12 weeks (Tables 1, 2, 3). PTNS was generally administered at 20 Hz, 0−10 mA, with a 200 µs pulse width, while TTNS settings were more variable. The most common settings observed with TTNS were 10 Hz (20% of studies) or 20 Hz (20% of studies), 0−10 mA (30% of studies), and a 200 µs pulse width (40% of studies). Some studies in the mixed NLUTD/NN LUTD group [18, 22] and the MNE group [24, 25] involved daily treatment with TTNS. TTNS was delivered at home in four studies [18, 22, 23, 26].

### 3.3 Comparisons

Studies including mixed NLUTD/NN LUTD were mostly prospective cohort studies, and no study had a comparison group (Table 1). The studies on MNE (Table 2) and NN LUTD (Table 3) included randomized controlled trials (RCTs) as well as cohort studies with and without comparison groups.

### 3.4 Outcomes

All three groups (mixed NLUTD/NN LUTD, MNE, and NN LUTD) included subjective outcomes such as reported improvement in symptoms, reported lack of leakage, degree of response, “cure,” and questionnaires on LUTS and QOL. Most studies also included objective outcomes such as uroflow parameters, post‐void residual (PVR), voided volumes, or urodynamic parameters. For most studies, follow‐up was performed at the end of the treatment course. The longest follow‐up in any study for each population was 3 years for the mixed NLUTD/NN LUTD group [19], 2 weeks post‐stimulation for the MNE group [26], and 2 years for the NN LUTD group [27].

### 3.5 Efficacy in Mixed Neurogenic/Non‐Neurogenic LUTD

Summarized results are presented in Table 4. Full results are presented in Supporting Information S1: Table 2. Taken together, PTNS has variable efficacy for improving LUTS in patients with mixed NLUTD/NN LUTD (0%−100% improvement over a variety of subjective outcomes; Table 4) [16, 19, 20, 21, 28], and TTNS appears to have at least some urinary symptom improvement for around 80% of patients [18, 22, 23]. There is conflicting data on whether PTNS is more effective in NLUTD or NN LUTD.

**Table 4:** Summarized results of studies on tibial nerve stimulation for mixed neurogenic/non‐neurogenic LUTD (seven studies; one purely neurogenic).

Study & TNS treatment modality	Subjective outcomes	Questionnaire scores	Objective outcomes	Quality score^a^
Capitanucci et al. 2009 PTNS	‐Non‐neurogenic: 78% improved ‐NLUTD: 14% improved ‐Cure at 2 years: 71% of DV, 41% of OAB ‐Chronic stimulation needed to maintain results: 29% of DV, 50% of OAB (NS)		‐Normalization of uroflow voided volume: 57% of DV, 20% of OAB ‐Normalization of uroflow PVR: 57% of DV, 25% of OAB	4
Cardona‐Grau et al. 2018 TTNS	−79% (22/28) subjectively improved −75% (21/28) subjectively complied with treatment	Change in DVSS score: ‐OAB group: −5.5 (95% CI 3−8, *p* = 0.004) ‐NLUTD group: not reported	−50% (14/28) had objective improvement based on “clinical parameters” ‐No significant changes in PVR in any group −75% (21/28) completed > 50% of treatments (measured by TENS unit)	4
Lecompte et al. 2015 TTNS	−83% dry at 6 months ‐All patients using anticholinergics stopped the medication	Improvements in the Schurch urinary incontinence score at 2 and 6 months		4
Roić et al. 2021 TTNS	−20% (8/39) had no symptom improvement −30.8% (12/39) had a better sensation of bladder fullness −15.4% (6/39) achieved urinary continence −10.2% (4/39) voided with less straining			4
DeGennaro et al. 2004 PTNS	‐OAB: 80% (8/10) improved ‐Incontinence associated with nighttime wetting: 44% (4/9) completely dry ‐UR: 71% (5/7) improved, 33% (1/3) dry ‐NLUTD: no significant improvement in symptoms	Transient pain during stimulation tended to decrease during sessions and remain stable/decreased across multiple sessions (Faces Pain Rating Scale, Children's Hospital of Eastern Ontario Pain Scale, Visual Analog Scale, Questionario Italiano del Dolore)	‐OAB: DO disappeared in children who became dry, low cystometric bladder capacity normalized in 63%, mean cystometric bladder capacity increased (NS) ‐UR: Significant PVR disappeared in 50%, mean Qmax and pressure at Qmax increased (NS) ‐NLUTD: Mean cystometric bladder capacity increased, mean PVR decreased (NS)	3
Ansari et al. 2020 PTNS	−54% (12/21) clinically improved ‐OAB: 58.3% (7/12) improved, 43% (3/7) completely cured ‐Underactive bladder: 56% (5/9) improved, 40% (2/5) completely cured		Brain activity on 18F‐FDG PET/CT: ‐Decreased uptake in “areas involved in the [sensation] of bladder filling” for OAB ‐Increased uptake in “areas involved in sensorimotor learning and the initiation of voiding” for underactive detrusor	1
Taverna et al. 2016 PTNS	−87.5% (7/8) had improvement in urgency and UI symptoms at 12 weeks −75% (3/4) of the non‐neurogenic group had disappearance of diurnal incontinence at 3 weeks ‐ All patients (4/4) in spina bifida neurogenic OAB group had complete disappearance of diurnal incontinence at 12 weeks and sustained improvement at 6−24 months		‐No changes in cystometric bladder capacity or detrusor overactivity on UDS ‐All three patients with improvement in urodynamic tests post‐treatment had maintenance of urodynamic results at 3‐year follow‐up ‐All patients (4/4) in spina bifida neurogenic OAB group had maintenance of urodynamic results at 6−24 months	0

Abbreviations: 18F‐FDG PET/CT, 18‐fluorodeoxyglucose positron emission tomography/computed tomography; DV, dysfunctional voiding; DVSS, dysfunctional voiding symptom score; NLUTD, neurogenic lower urinary tract dysfunction; NS, not statistically significant; OAB, overactive bladder; PTNS, percutaneous tibial nerve stimulation; PVR, post‐void residual; Qmax, maximum flow; TNS, tibial nerve stimulation; TTNS, transcutaneous tibial nerve stimulation; UDS, urodynamic studies; UR, urinary retention.

^a^ Quality score based on the Mixed Methods Assessment Tool: high (5/5 criteria met); moderate (3/5 or 4/5 criteria met); poor (0−2/5 criteria met).

Among the highest‐quality studies (MMAT quality score 4), PTNS was less effective in NLUTD than NN LUTD [20], and the majority of NLUTD patients undergoing TTNS had subjective improvements in LUTS [18, 22, 23]. However, all studies were quantitative descriptive studies without comparison groups, and diverse and non‐standardized outcomes made study comparisons challenging. The risk of nonresponse bias was unable to be assessed for all studies. Based on the GRADE approach, the certainty of evidence for the outcome of LUTS improvement was low (Supporting Information S1: Table 5).

#### 3.5.1 Factors Affecting Outcomes

While PTNS was performed for 30 min weekly for 12 weeks, TTNS was performed anywhere from daily to twice weekly. Good treatment compliance was noted with home TTNS, with patients completing on average 87% of their assigned treatment and 79% of patients completing over 50% of treatments [23]. The increased utilization of TTNS may be related to the high improvement rate.

### 3.6 Efficacy in MNE

Summarized results are presented in Table 5. Full results are presented in Supporting Information S1: Table 3. Taken together, most studies showed at least a mild improvement in nocturnal enuresis. The number of wet nights decreased by 1−2 over 1−2 weeks with PTNS [29] and TTNS [26, 30]. Other studies reported “improvement” in 60%−80% of patients without specifying the number of wet nights [31, 32], and a single study reported no improvement compared to sham treatment [25]. TTNS was slightly more effective than a bedwetting alarm [33], while PTNS had comparable efficacy to desmopressin [34].

**Table 5:** Summarized results of studies on tibial nerve stimulation for nocturnal enuresis (eight studies).

Study & TNS treatment modality	Subjective outcomes	Questionnaire scores	Objective outcomes	Quality score^a^
Ferroni et al. 2017 TTNS	−72.7% (16/22) had at least 1 fewer wet night during the treatment period Mean (SD) total wet nights over 2 weeks: −9 (4) pre‐stimulation −6.8 (4.8) during stimulation (*p* < 0.01 vs. pre) −7.2 (5.0) post‐stimulation (*p* = 0.02 vs. pre)	‐No significant differences in QOL (PedsQL) or LUTS (Vancouver NLUTD/DES questionnaire) between the periods before, during, or after stimulation overall ‐ Vancouver NLUTD/DES questionnaire score improved for responders (mean 13.9 before, 11.6 during, and 10.5 after stimulation)	Mean age (range): Responders: 12 (8−16) Nonresponders: 8.7 (7−10) (*p* < 0.01)	5
Raheem et al. 2013 PTNS	‐PTNS had higher rates of full response (29% vs. 0%) and partial response (50% vs. 14%) versus sham ‐Greater decrease in number of wet nights over 2 weeks for PTNS versus sham (*p* = 0.041) ‐PTNS: mean (SD) 4.7 (1.3) to 2.6 (2.2) Sham: mean (SD) 5.1 (1.4) to 4.7 (2.1)		‐Volume at first desire, volume at strong desire, maximum voided volume, and maximum cystometric capacity increased significantly for PTNS but not sham ‐Detrusor overactivity decreased significantly for PTNS but not sham	4
Elshafey et al. 2015 TTNS	−87.5% recovery rate with TTNS versus 75% with bedwetting alarm (*p* = 0.04) ‐Mean (SD) frequency of nocturnal enuresis (over unknown time period) decreased from 5.90 (0.41) to 1.20 (0.14) with TTNS (*p* = 0.001), versus 6.30 (0.23) to 3.40 (0.35) with bedwetting alarm (*p* = 0.001)	1. Mean (SD) KIDSCREEN‐10 Index score (health‐related quality of life) increased from 1.80 (0.04) to 3.90 (0.73) with TTNS (*p* = 0.001), versus 1.56 (0.09) to 2.80 (0.05) (*p* = 0.001) with bedwetting alarm	Mean (SD) maximum voided volume (mL) increased from 160 (15) to 192 (12) for TTNS (*p* = 0.001), versus 155 (14) to 176 (10) for bedwetting alarm (*p* = 0.001)	2
Amar et al. 2020 PTNS	Both PTNS and desmopressin groups had improved frequency of nocturnal enuresis (statistically significant, over unknown time period) and relapse after 1 month. No significant differences between groups			2
AlZamil et al. 2020 TTNS	The average frequency of wet nights per week was 4.7 ± 1.4 in both groups pre‐treatment. Post treatment: 3.5 ± 1.6 in high‐frequency/low‐amplitude group, versus 1.4 ± 1.1 in low‐frequency/high‐amplitude group (1.7x more effective)		‐The high‐frequency/low‐amplitude group had no significant changes in theta index on EEG activity ‐Low‐frequency/high‐amplitude group had a 39% decrease in theta index	1
Vasudevan et al. 2018 TTNS	TTNS group had mean (SD) decrease in wet nights −4.2% (5.9%) (over unknown time period), more than sham and PTENS groups, but not statistically significant (*p* = 0.128)	PIN‐Q score: improved in all groups, but no significant differences between PTNS/PTENS/sham groups at months 1 (*p* = 0.446), 2 (*p* = 0.858), or 3 (*p* = 0.737).		1
Aboul Ela et al. 2014 PTNS	60% (12/20) improved		60% (12/20) had improvement of all urodynamic parameters with an increase in bladder capacity and disappearance of detrusor instability	0
Perez‐Martinez et al. 2020 PTNS	Mean (SD) patient improvement (defined as “dry nights,” over an unknown time period): 80% (7.5%) for PTNS 3.7% (1.2%) for sham (*p* < 0.001) Mean (SD) dry nights 1 month after treatment: 92.5% (1.4%) for PTNS 10% (3.5%) for sham (*p* < 0.001)			0

Abbreviations: DES, dysfunctional elimination syndrome; NLUTD, non‐neurogenic lower urinary tract dysfunction; PedsQL, pediatric quality of life inventory; PIN‐Q, pediatric incontinence questionnaire quality of life; PTENS, parasacral transcutaneous electrical nerve stimulation; PTNS, percutaneous tibial nerve stimulation; QOL, quality of life; SD, standard deviation; TNS, tibial nerve stimulation; TTNS, transcutaneous tibial nerve stimulation.

^a^ Quality score based on the Mixed Methods Assessment Tool: high (5/5 criteria met); moderate (3/5 or 4/5 criteria met); poor (0−2/5 criteria met).

Among the highest‐quality studies (MMAT quality score 4−5), children treated with PTNS [29] and TTNS [26] had, on average, two fewer wet nights over a 2‐week period. PTNS also improved maximum voided volume and cystometric capacity compared to sham treatment [29]. Interestingly, neither responders to TTNS nor participants as a whole had significant improvements in their QOL [26]. The overall quality of evidence was low (six studies), with only one moderate‐quality RCT [29] and one high‐quality quantitative descriptive study [26]. The remaining studies did not consistently report complete outcome data, participant adherence, blinding of outcome assessors in RCTs, or consideration of confounding factors. The certainty of evidence for the outcome of frequency of wet nights was low (Supporting Information S1: Table 5).

#### 3.6.1 Factors Affecting Outcomes

One study on TTNS reported that low‐frequency settings (1 Hz, 200 µs) were more effective than high‐frequency settings (100 Hz, 100 µs), although the baseline number of wet nights for each group was not reported [30]. Compliance with treatment was not analyzed. In one study, patients who responded to TTNS had significantly fewer wet nights at baseline during the 2‐week pre‐stimulation period (mean 7.9 vs. 12) and were older (mean age 12 vs. 8.7 years) [26] than nonresponders, suggesting that older age and/or milder symptoms may be associated with better response.

### 3.7 Efficacy in Non‐Neurogenic LUTD

Summarized results are presented in Table 6. Full results are presented in Supporting Information S1: Table 4. The overall quality of evidence was mixed, including studies of low (*n* = 2), medium (*n* = 2), and high (*n* = 4) quality. Some RCTs did not report blinding of outcome assessors or participant adherence, and some quantitative descriptive studies did not provide adequate information to determine the risk of nonresponse bias. For all studies, compliance with treatment was not analyzed.

**Table 6:** Summarized results of studies on tibial nerve stimulation for non‐neurogenic LUTD (eight studies).

Study & TNS treatment modality	Subjective outcomes	Questionnaire scores	Objective outcomes	Quality score^a^
Barroso Jr. et al. 2013 PTNS	Parent‐reported full response: 9% for PTNS versus 70% for PTENS (*p* = 0.02) After treatment, persistent urgency in 23% for PTNS versus 12% for PTENS (NS), persistent diurnal incontinence in 41% for PTNS versus 20% for PTENS (NS), persistent enuresis in 64% of PTNS versus 59% for PTENS (NS)	Mean ± SD DVSS decreased from 10.1 ± 5.0 to 2.5 ± 3.1 for PTNS, versus 10.6 ± 5.0 to 2.3 ± 3.1 for PTENS (*p* = 0.55)		5
Boudaoud et al. 2015 TTNS	Felt stimulation: TTNS 85%, sham 71%.	Mean (SD) urinary score (0 best, 13 worst): TTNS 5.72 (2.14) to 6.18 (4.55) Sham 5.66 (3.08) to 5.0 (3.87) Results based on change in urinary score: Very good: TTNS 45% (5/11), sham 66% (6/9) Medium: TTNS 9% (1/11), sham 0% Poor: TTNS 45% (5/11), sham 33% (3/9)	TTNS had improvement in mean voided volume during urgency episodes (184−265 mL, *p* = 0.002), mean maximum cystomanometry volume (215−274 mL, *p* = 0.024), mean volume during first overactive detrusor contraction (48−174 mL, *p* = 0.001), mean maximum detrusor pressure (61−46 cm H_2_O, *p* = 0.042) Sham group had no significant changes in any parameter	5
DeWall et al. 2022 PTNS	10% complete response (100% cure) 32% partial response (50%−99% improvement) 58% no response (0%−49% improvement)	‐Median child‐reported PIN‐Q score decreased (i.e., better QOL) from 25 to 19 (*p* = 0.001) ‐Median parent‐reported PIN‐Q score decreased (i.e., better QOL) from 26 to 22 (*p* = 0.001)	‐Median average voided volume (mL) increased from 114 to 139 (*p* = 0.000) ‐Median maximum voided volume (mL) increased from 192 to 205 (*p* = 0.025)	5
Mendes et al. 2016 PTNS	33% had improved continence (*p* = 1) 50% had improvement in UTIs (*p* = 0.1) 50% had less abdominal straining (*p* = 0.2)		‐Improvements in mean ± SD flow time (59 ± 34 s to 39 ± 15 s; *p* = 0.05), PVR (146.6 ± 176.1 mL to 39.6 ± 49.9; *p* = 0.01), mean voided volume (246.8−284.6 mL; *p* = 0.2) −63% had improved flow pattern (*p* = 0.0001) −71% of patients had improved PVR (*p* = 0.0002)	5
Hoebeke et al. 2002 PTNS	−84% (27/32) responded to treatment ‐Significant improvement in disturbed voiding frequency (< 4 or > 8 voids per day): 19/31 before treatment, 3/31 after treatment (*p* < 0.001) ‐No significant improvements in urgency or daytime incontinence ‐No complications		‐Mean maximum bladder capacity increased from 185 to 279 cc (*p* < 0.001). ‐Abnormal flow curve decreased from 21/31 to 12/31 patients (*p* = 0.004)	4
Jafarov et al. 2021 TTNS	−2 weeks post‐treatment: Cured: TTNS 50% (10/20), sham 20% (2/10) Improved: TTNS 20% (4/20), sham 40% (4/10) No change: TTNS 30% (6/20), sham 40% (4/10) ‐Similar 2‐year results ‐No significant differences in daytime episodes of urgency, daytime episodes of incontinence, or nocturnal episodes of incontinence before or after treatment (long‐term or short‐term) for TTNS or sham	‐Both TTNS and sham: significant differences in DVISS scores (overall, QOL, daytime) before and after treatment, but not long‐term after treatment ‐No significant differences in nighttime DVISS score before and after treatment	No difference in urinary NGF, TGFβ‐1, and TIMP‐2 levels before or after treatment for TTNS or sham	3
Ibrahim et al. 2019 PTNS	−60% (12/20) requested to continue treatment (“success”) ‐Improvements in daytime frequency (60%, 12/20), urgency (55%, 11/20), nocturnal enuresis (63%, 10/16), urge incontinence (53%, 8/15)		‐Bladder capacity (mean ± SD) increased from 184.5 ± 59.14 to 259.5 ± 77.22 (*p* = 0.001) −70% (14/20) had disappearance of involuntary detrusor contractions on UDS ‐Improvements in compliance in 57% (4/7) of patients with mild hypocompliance, 20% (1/5) of patients with moderate hypocompliance	2
Patidar et al. 2015 TTNS	−67% (14/21) cure for TTNS versus 0% (0/16) for sham −24% significantly improved for TTNS versus 6% for sham −9% partial response for TTNS versus 19% for sham −0% no response for TTNS versus 75% for sham		For TTNS, significant improvements in mean average voided volume (68−89 mL; *p* = 0.001), mean maximum voided volume (116−190 mL; *p* = 0.01), and mean number of voids (11−7; *p* = 0.001). No significant changes for sham	2

Abbreviations: DVSS, dysfunctional voiding symptom score; DVISS, dysfunctional voiding and incontinence symptom score; PIN‐Q, pediatric incontinence questionnaire; PTENS, parasacral transcutaneous electrical nerve stimulation; PTNS, percutaneous tibial nerve stimulation; PVR, post‐void residual; QOL, quality of life; SD, standard deviation; TNS, tibial nerve stimulation; TTNS, transcutaneous tibial nerve stimulation; UTI, urinary tract infection.

^a^ Quality score based on the Mixed Methods Assessment Tool: high (5/5 criteria met); moderate (3/5 or 4/5 criteria met); poor (0−2/5 criteria met).

#### 3.7.1 OAB

Taken together, about 60%−70% of patients with OAB treated with PTNS or TTNS had at least some improvement, although cure rates were variable [35, 36, 37, 38]. Among the highest‐quality studies (MMAT quality score 5), TTNS improved voided volume and bladder capacity on urodynamics, but symptom improvement was mixed (45% had a “very good” response, 45% had a “poor” response; the remainder had a “medium” response) [36]. PTENS more often led to resolution of symptoms than PTNS in one study [39]. The authors postulate that the effect of PTENS may have been due to direct stimulation of the sacral nerves, a higher number of treatments (20 sessions, vs. 12 for PTNS), or the lower frequency and greater pulse width used for PTENS. The certainty of evidence for the outcome of OAB was low (Supporting Information S1: Table 5).

#### 3.7.2 Dysfunctional Voiding

Taken together, about 50% of patients with dysfunctional voiding treated with PTNS had improved flow patterns, although there were minimal to no improvements in continence [40, 41]. Both studies were medium‐to‐high quality (MMAT score 4−5) and involved 30 min of weekly stimulation for a similar duration (10 [41] vs. 12 weeks [40]) in patients with prior noninvasive treatments. The certainty of evidence for the outcome of dysfunctional voiding was low (Supporting Information S1: Table 5).

#### 3.7.3 Non‐MNE

Taken together, there is insufficient data at this time to comment on the efficacy of TNS for non‐MNE. In one study, 64% of patients had persistent symptomatic enuresis after PTNS, although the degree of enuresis before and after treatment was not reported [39]. In another study, 63% of patients had improvements in nocturnal enuresis that were not quantified [37]. Studies varied in quality (MMAT score 2−5), but enuresis was not a primary outcome of either study.

#### 3.7.4 Other NN LUTD

Taken together, the rate of complete cure (10%−50%) and improvement without complete cure (20%−32%) for unspecified NN LUTD were variable for TNS [27, 42]. Studies were low‐to‐medium quality (MMAT score 2−3). A single qualitative study found that parents and children chose TNS to avoid medication or surgery [42]. Home treatment was preferred to avoid traveling and having to leave school. Pain with needle insertion was generally absent to mild, and distraction techniques were felt to be helpful.

### 3.8 Adverse Effects

Studies that reported adverse effects noted that these were few or absent [18, 32, 37, 40]. Transient mild pain at the needle insertion site was noted to decrease during stimulation based on the VAS [37] in NN LUTD. In a mixed NLUTD/NN LUTD cohort, patients did not demonstrate improved pain over 12 PTNS sessions based on the Faces Pain Rating Scale or VAS, but there was a slight improvement in pain based on the Questionario Italiano del Dolore, and fewer children were noted to cry or complain during treatment as the 12‐week treatment period progressed [21].

## 4 Discussion

In this systematic review of TNS in children with LUTS and LUTD, we found that TNS generally yielded objective or urodynamic improvements with some correlation to improved symptoms and QOL. Non‐neurogenic patients generally experienced more consistent improvements in symptoms, bladder function, and urodynamic parameters with PTNS than neurogenic patients. For nocturnal enuresis, PTNS led to a mild reduction in wet nights and increased bladder capacity, which were sometimes reflected by improvements in QOL. For NN LUTD, PTNS, and TTNS, both yielded improvements in symptoms and urodynamic parameters, although studies on PTNS lacked sham controls, and studies on TTNS did not have consistent efficacy compared to sham treatment. While urodynamic changes do not always correlate with clinical or subjective improvement, and symptom improvements do not always translate to better QOL, there overall appears to be at least a mild benefit to TNS treatment, although overall certainty of evidence was low. More sham‐controlled studies directly comparing PTNS, TTNS, and other treatments are needed to determine the relative efficacy of these approaches and optimize patient selection.

Similar to our findings in children, TNS in adults has demonstrated improvements in both urodynamic parameters and patient‐reported LUTS. A multicenter, sham‐controlled RCT found that for adults with OAB, PTNS significantly improved bladder symptoms, voiding diary parameters, and QOL compared to sham treatment [13]. A systematic review assessing the safety and efficacy of PTNS and TTNS in adults with NLUTD reported treatment‐related improvements in urodynamic parameters such as maximum cystometric capacity, maximum detrusor pressure during filling, and bladder volume at first involuntary contraction [14]. These studies also reported improvements in the frequency of voids, episodes of incontinence, and PVR [14]. Similar to studies in the pediatric population, the studies reviewed in adults were limited by a high risk of bias and inadequate controlling for confounding factors.

Similar to TNS, the literature for PTENS in pediatric LUTD appears mixed regarding efficacy. A systematic review and meta‐analysis found that for pediatric LUTD, PTENS improved both LUTS (incontinence episodes, urgency, voiding frequency) and objective measures (voided volumes, bladder capacity, and function) [43]. Another systematic review and meta‐analysis assessing outcomes of PTENS for nocturnal enuresis found mixed results regarding symptomatic improvement and objective measures [44]. Across four RCTs, only 11% of children with nocturnal enuresis achieved a full response after PTENS, defined as complete dryness or elimination of enuresis episodes. There were no significant differences between PTENS and control treatments including sham, behavioral therapies, and biofeedback. Direct comparison between TNS and PTENS in future studies may help clarify the role of each treatment.

This review has several limitations and strengths. The overall quality of evidence remains low to moderate. Several studies reported “improvement” without a defining threshold. Heterogeneity in study designs, non‐standardized outcomes, patient populations, and sample sizes makes direct comparisons between studies challenging. This current review systematically reviews studies on both NLUTD and NN LUTD. This approach addresses limitations of existing reviews on TNS in children, which were either non‐systematic, outdated, or limited in scope, focusing primarily on NN LUTD.

Our findings highlight future directions for high‐quality research in this area. Future studies should standardize outcomes using validated questionnaires and provide more data on maintenance therapies, duration of response, and long‐term outcomes. Sham‐controlled trials with direct comparisons between PTNS/TTNS and other treatments, such as PTENS or desmopressin for nocturnal enuresis, would provide important context. Larger, well‐characterized cohorts can help inform patient selection. The efficacy of TNS in NLUTD needs to be further explored. Lastly, understanding patient and parent experiences and preferences will help inform counseling on individualized treatment options for LUTS and LUTD.

## 5 Conclusions

In children with LUTS and LUTD, TNS delivered both percutaneously and transcutaneously often improves objective bladder dysfunction parameters with some correlation to improved symptoms and QOL. TNS likely offers at least a mild improvement in nocturnal enuresis. While improvements may be more consistently seen in children with NN LUTD compared to NLUTD, evidence remains limited by small sample sizes, absence of comparison groups in several studies, and variability in reported outcomes. Future research should focus on conducting larger, well‐controlled trials to further define the role of TNS in managing pediatric LUTS and LUTD.

## Conflicts of Interest

The authors declare no conflicts of interest.

## Supporting information

TNS Supplemental Material 1.

Supplemental Table 1. Full Mixed Methods Assessment Tool quality assessment ratings for included studies.

Supplemental Table 5. GRADE approach for certainty assessment of outcomes.

Supplemental Table 2. Full results of studies on tibial nerve stimulation for mixed neurogenic/non‐neurogenic LUTD (7 studies; 1 purely neurogenic). Supplemental Table 3. Full results of studies on tibial nerve stimulation for nocturnal enuresis (8 studies). Supplemental Table 4. Full results of studies on tibial nerve stimulation for non‐neurogenic LUTD (8 studies).

## Data Availability

Data sharing is not applicable to this article as no data sets were generated or analysed during the current study.
