# Supplementary material for: Systematic Review of Percutaneous and Transcutaneous Posterior Tibial Neurostimulation for Lower Urinary Tract Symptoms & Lower Urinary Tract Dysfunction in Children
Source: Neurourol Urodyn. 2026 Mar 15;45(4):774–93. doi: 10.1002/nau.70264 (PMC13054631; doi:10.1002/nau.70264)
Supplement: Supplementary file 1 — TNS Supplemental Material 1. [file NAU-45-774-s004.docx]

Supplemental Material 1. Complete search strategy for PubMed, CENTRAL, EMBASE, and Scopus.

- PubMed search, conducted on May 11, 2023 – search saved

1. PTNS concept: 3,216 hits

"tibial nerve stimulat*"[tiab] OR "PTNS"[tiab] OR (("Tibial Nerve"[mesh] OR "tibial nerv*"[tiab] OR "sacral nerv*"[tiab] OR "nervus tibialis"[tiab] OR "parasacral"[tiab] OR "sacral"[tiab]) AND ("Transcutaneous Electric Nerve Stimulation"[mesh] OR "transcutaneous electric nerve stimulat*"[tiab] OR "percutaneous electric nerve stimulat*"[tiab] OR "electric stimulation therapy"[mesh] OR "electric stimulation therap*"[tiab] OR "neurostimulat*"[tiab] OR "Transcutaneous Electrical Stimulat*"[tiab] OR "Transdermal Electrostimulat*"[tiab] OR "Transcutaneous Electrical Nerve Stimulat*"[tiab] OR "Transcutaneous Nerve Stimulat*"[tiab] OR "Percutaneous Neuromodulation Therap*"[tiab] OR "Percutaneous Electrical Neuromodulat*"[tiab] OR "Percutaneous Electrical Neuromodulat*"[tiab] OR "percutaneous electrical stimulat*"[tiab] OR "transcutaneous electrical nerve stimulat*"[tiab] OR "Transcutaneous neuromodulat*"[tiab]))

1. Children concept: 3,200,750 hits

"Child"[Mesh] OR "Pediatrics"[Mesh] OR "child"[tiab] OR "child*"[tiab] OR "schoolchild*"[tiab] OR "schoolchild"[tiab] OR "school child"[tiab] OR "school child*"[tiab] OR "kid"[tiab] OR "kids"[tiab] OR "toddler*"[tiab] OR "adolescent"[tiab] OR "adoles*"[tiab] OR "teen*"[tiab] OR "boy*"[tiab] OR "girl*"[tiab] OR "minors"[tiab] OR "minors*"[tiab] OR "underag*"[tiab] OR "under ag*"[tiab] OR "juvenil*"[tiab] OR "youth*"[tiab] OR "kindergar*"[tiab] OR "puberty"[tiab] OR "puber*"[tiab] OR "pubescen*"[tiab] OR "prepubescen*"[tiab] OR "prepuberty*"[tiab] OR "pediatric*"[tiab] OR "paediatric*"[tiab] OR "peadiatric*"[tiab] OR "schools"[tiab] OR "nursery school*"[tiab] OR "preschool*"[tiab] OR "pre school*"[tiab] OR "primary school*"[tiab] OR "secondary school*"[tiab] OR "elementary school*"[tiab] OR "elementary school"[tiab] OR "high school*"[tiab] OR "highschool*"[tiab] OR "school age"[tiab] OR "schoolage"[tiab] OR "school age*"[tiab] OR "schoolage*"[tiab]

1. Lower Urinary Tract Dysfunction concept: 711,407 hits

"urinary tract*"[tiab] OR "urinary symptom*"[tiab] OR "urodynamic test*"[tiab] OR "Lower Urinary Tract Symptoms"[Mesh] OR "urinary bladder, overactive"[mesh] OR "Overactive Bladder"[tiab] OR "Overactive Urinary Bladder"[tiab] OR "Overactive Detrusor*"[tiab] OR "urinary tract"[mesh] OR "urinary incontinence"[mesh] OR "urinary incontinence"[tiab] OR "urinary bladder diseases"[mesh] OR "bladder disease*"[tiab] OR "urination disorders"[mesh] OR "urination disorder*"[tiab] OR "urodynamics"[mesh] OR "urodynamic*"[tiab] OR "urodynamic parameter*"[tiab] OR "urge incontinence"[tiab] OR "hyperactive bladder"[tiab] OR "enuresis"[tiab] OR "urinary urge incontinence"[tiab] OR "urge syndrome"[tiab]

1. Combination #1 AND #2 AND #3: 119 hits
   **(("tibial nerve stimulat*"[tiab] OR "PTNS"[tiab] OR (("Tibial Nerve"[mesh] OR "tibial nerv*"[tiab] OR "sacral nerv*"[tiab] OR "nervus tibialis"[tiab] OR "parasacral"[tiab] OR "sacral"[tiab]) AND ("Transcutaneous Electric Nerve Stimulation"[mesh] OR "transcutaneous electric nerve stimulat*"[tiab] OR "percutaneous electric nerve stimulat*"[tiab] OR "electric stimulation therapy"[mesh] OR "electric stimulation therap*"[tiab] OR "neurostimulat*"[tiab] OR "Transcutaneous Electrical Stimulat*"[tiab] OR "Transdermal Electrostimulat*"[tiab] OR "Transcutaneous Electrical Nerve Stimulat*"[tiab] OR "Transcutaneous Nerve Stimulat*"[tiab] OR "Percutaneous Neuromodulation Therap*"[tiab] OR "Percutaneous Electrical Neuromodulat*"[tiab] OR "Percutaneous Electrical Neuromodulat*"[tiab] OR "percutaneous electrical stimulat*"[tiab] OR "transcutaneous electrical nerve stimulat*"[tiab] OR "Transcutaneous neuromodulat*"[tiab]))) AND ("Child"[Mesh] OR "Pediatrics"[Mesh] OR "child"[tiab] OR "child*"[tiab] OR "schoolchild*"[tiab] OR "schoolchild"[tiab] OR "school child"[tiab] OR "school child*"[tiab] OR "kid"[tiab] OR "kids"[tiab] OR "toddler*"[tiab] OR "adolescent"[tiab] OR "adoles*"[tiab] OR "teen*"[tiab] OR "boy*"[tiab] OR "girl*"[tiab] OR "minors"[tiab] OR "minors*"[tiab] OR "underag*"[tiab] OR "under ag*"[tiab] OR "juvenil*"[tiab] OR "youth*"[tiab] OR "kindergar*"[tiab] OR "puberty"[tiab] OR "puber*"[tiab] OR "pubescen*"[tiab] OR "prepubescen*"[tiab] OR "prepuberty*"[tiab] OR "pediatric*"[tiab] OR "paediatric*"[tiab] OR "peadiatric*"[tiab] OR "schools"[tiab] OR "nursery school*"[tiab] OR "preschool*"[tiab] OR "pre school*"[tiab] OR "primary school*"[tiab] OR "secondary school*"[tiab] OR "elementary school*"[tiab] OR "elementary school"[tiab] OR "high school*"[tiab] OR "highschool*"[tiab] OR "school age"[tiab] OR "schoolage"[tiab] OR "school age*"[tiab] OR "schoolage*"[tiab]) AND ("urinary tract*"[tiab] OR "urinary symptom*"[tiab] OR "urodynamic test*"[tiab] OR "Lower Urinary Tract Symptoms"[Mesh] OR "urinary bladder, overactive"[mesh] OR "Overactive Bladder"[tiab] OR "Overactive Urinary Bladder"[tiab] OR "Overactive Detrusor*"[tiab] OR "urinary tract"[mesh] OR "urinary incontinence"[mesh] OR "urinary incontinence"[tiab] OR "urinary bladder diseases"[mesh] OR "bladder disease*"[tiab] OR "urination disorders"[mesh] OR "urination disorder*"[tiab] OR "urodynamics"[mesh] OR "urodynamic*"[tiab] OR "urodynamic parameter*"[tiab] OR "urge incontinence"[tiab] OR "hyperactive bladder"[tiab] OR "enuresis"[tiab] OR "urinary urge incontinence"[tiab] OR "urge syndrome"[tiab]))**

Note: the final #4 search included the 18 articles in the provided benchmark set.

- CENTRAL search, conducted on May 11, 2023 – search saved

1. PTNS concept: 596 hits

("tibial nerve" NEXT stimulat*):ti,ab OR PTNS:ti,ab OR (([mh "Tibial Nerve"] OR ("tibial" NEXT nerv*):ti,ab OR ("sacral" NEXT nerv*):ti,ab OR "nervus tibialis":ti,ab OR parasacral:ti,ab OR sacral:ti,ab) AND ([mh "Transcutaneous Electric Nerve Stimulation"] OR ("transcutaneous electric nerve" NEXT stimulat*):ti,ab OR ("percutaneous electric nerve" NEXT stimulat*):ti,ab OR [mh "electric stimulation therapy"] OR ("electric stimulation" NEXT therap*):ti,ab OR neurostimulat*:ti,ab OR ("Transcutaneous Electrical" NEXT Stimulat*):ti,ab OR ("Transdermal" NEXT Electrostimulat*):ti,ab OR ("Transcutaneous Electrical Nerve" NEXT Stimulat*):ti,ab OR ("Transcutaneous Nerve" NEXT Stimulat*):ti,ab OR ("Percutaneous Neuromodulation" NEXT Therap*):ti,ab OR ("Percutaneous Electrical" NEXT Neuromodulat*):ti,ab OR ("Percutaneous Electrical" NEXT Neuromodulat*):ti,ab OR ("percutaneous electrical" NEXT stimulat*):ti,ab OR ("transcutaneous electrical nerve" NEXT stimulat*):ti,ab OR ("Transcutaneous" NEXT neuromodulat*):ti,ab))

2. Children concept: 216,359 hits

[mh Child] OR [mh Pediatrics] OR child:ti,ab OR child*:ti,ab OR schoolchild*:ti,ab OR schoolchild:ti,ab OR "school child":ti,ab OR ("school" NEXT child*):ti,ab OR kid:ti,ab OR kids:ti,ab OR toddler*:ti,ab OR adolescent:ti,ab OR adoles*:ti,ab OR teen*:ti,ab OR boy*:ti,ab OR girl*:ti,ab OR minors:ti,ab OR minors*:ti,ab OR underag*:ti,ab OR ("under" NEXT ag*):ti,ab OR juvenil*:ti,ab OR youth*:ti,ab OR kindergar*:ti,ab OR puberty:ti,ab OR puber*:ti,ab OR pubescen*:ti,ab OR prepubescen*:ti,ab OR prepuberty*:ti,ab OR pediatric*:ti,ab OR paediatric*:ti,ab OR peadiatric*:ti,ab OR schools:ti,ab OR ("nursery" NEXT school*):ti,ab OR preschool*:ti,ab OR ("pre" NEXT school*):ti,ab OR ("primary" NEXT school*):ti,ab OR ("secondary" NEXT school*):ti,ab OR ("elementary" NEXT school*):ti,ab OR "elementary school":ti,ab OR ("high" NEXT school*):ti,ab OR highschool*:ti,ab OR "school age":ti,ab OR schoolage:ti,ab OR ("school" NEXT age*):ti,ab OR schoolage*:ti,ab

3. Lower Urinary Tract Dysfunction concept: 33,126 hits

("urinary" NEXT tract*):ti,ab OR ("urinary" NEXT symptom*):ti,ab OR ("urodynamic" NEXT test*):ti,ab OR [mh "Lower Urinary Tract Symptoms"] OR [mh "urinary bladder, overactive"] OR "Overactive Bladder":ti,ab OR "Overactive Urinary Bladder":ti,ab OR ("Overactive" NEXT Detrusor*):ti,ab OR [mh "urinary tract"] OR [mh "urinary incontinence"] OR "urinary incontinence":ti,ab OR [mh "urinary bladder diseases"] OR ("bladder" NEXT disease*):ti,ab OR [mh "urination disorders"] OR ("urination" NEXT disorder*):ti,ab OR [mh urodynamics] OR urodynamic*:ti,ab OR ("urodynamic" NEXT parameter*):ti,ab OR "urge incontinence":ti,ab OR "hyperactive bladder":ti,ab OR enuresis:ti,ab OR "urinary urge incontinence":ti,ab OR "urge syndrome":ti,ab

4. Combination #1 AND #2 AND #3: 47 hits

- EMBASE search, conducted on May 11, 2023 – search saved

1. PTNS concept: 7,272 hits

'tibial nerve stimulat*':ti,ab OR ptns:ti,ab OR (('tibial nerve'/exp OR 'tibial nerve' OR 'tibial nerv*':ti,ab OR 'sacral nerv*':ti,ab OR 'nervus tibialis':ti,ab OR parasacral:ti,ab OR sacral:ti,ab) AND ('transcutaneous electric nerve stimulation'/exp OR 'transcutaneous electric nerve stimulation' OR 'transcutaneous electric nerve stimulat*':ti,ab OR 'percutaneous electric nerve stimulat*':ti,ab OR 'electric stimulation therapy'/exp OR 'electric stimulation therapy' OR 'electric stimulation therap*':ti,ab OR neurostimulat*:ti,ab OR 'transcutaneous electrical stimulat*':ti,ab OR 'transdermal electrostimulat*':ti,ab OR 'transcutaneous nerve stimulat*':ti,ab OR 'percutaneous neuromodulation therap*':ti,ab OR 'percutaneous electrical neuromodulat*':ti,ab OR 'percutaneous electrical stimulat*':ti,ab OR 'transcutaneous electrical nerve stimulat*':ti,ab OR 'transcutaneous neuromodulat*':ti,ab))

2. Children concept: 4,531,359 hits

'child'/exp OR 'pediatrics'/exp OR child:ti,ab OR child*:ti,ab OR schoolchild*:ti,ab OR schoolchild:ti,ab OR 'school child':ti,ab OR 'school child*':ti,ab OR kid:ti,ab OR kids:ti,ab OR toddler*:ti,ab OR adolescent:ti,ab OR adoles*:ti,ab OR teen*:ti,ab OR boy*:ti,ab OR girl*:ti,ab OR minors:ti,ab OR minors*:ti,ab OR underag*:ti,ab OR 'under ag*':ti,ab OR juvenil*:ti,ab OR youth*:ti,ab OR kindergar*:ti,ab OR puberty:ti,ab OR puber*:ti,ab OR pubescen*:ti,ab OR prepubescen*:ti,ab OR prepuberty*:ti,ab OR pediatric*:ti,ab OR paediatric*:ti,ab OR peadiatric*:ti,ab OR schools:ti,ab OR 'nursery school*':ti,ab OR preschool*:ti,ab OR 'pre school*':ti,ab OR 'primary school*':ti,ab OR 'secondary school*':ti,ab OR 'elementary school*':ti,ab OR 'elementary school':ti,ab OR 'high school*':ti,ab OR highschool*:ti,ab OR 'school age':ti,ab OR schoolage:ti,ab OR 'school age*':ti,ab OR schoolage*:ti,ab

3. Lower Urinary Tract Dysfunction concept: 1,046,232 hits

'urinary tract*':ti,ab OR 'urinary symptom*':ti,ab OR 'urodynamic test*':ti,ab OR 'lower urinary tract symptoms'/exp OR 'urinary bladder, overactive'/exp OR 'overactive bladder':ti,ab OR 'overactive urinary bladder':ti,ab OR 'overactive detrusor*':ti,ab OR 'urinary tract'/exp OR 'urinary incontinence'/exp OR 'urinary incontinence':ti,ab OR 'urinary bladder diseases'/exp OR 'bladder disease*':ti,ab OR 'urination disorders'/exp OR 'urination disorder*':ti,ab OR 'urodynamics'/exp OR urodynamic*:ti,ab OR 'urodynamic parameter*':ti,ab OR 'urge incontinence':ti,ab OR 'hyperactive bladder':ti,ab OR enuresis:ti,ab OR 'urinary urge incontinence':ti,ab OR 'urge syndrome':ti,ab

4. Combination #1 AND #2 AND #3: 253 hits

- Scopus search, conducted on May 11, 2023 – search saved

1. PTNS concept: 3,182 hits

TITLE-ABS ( "tibial nerve stimulat*" ) OR TITLE-ABS ( ptns ) OR ( ( INDEXTERMS ( "Tibial Nerve" ) OR TITLE-ABS ( "tibial nerv*" ) OR TITLE-ABS ( "sacral nerv*" ) OR TITLE-ABS ( "nervus tibialis" ) OR TITLE-ABS ( parasacral ) OR TITLE-ABS ( sacral ) ) AND ( INDEXTERMS ( "Transcutaneous Electric Nerve Stimulation" ) OR TITLE-ABS ( "transcutaneous electric nerve stimulat*" ) OR TITLE-ABS ( "percutaneous electric nerve stimulat*" ) OR INDEXTERMS ( "electric stimulation therapy" ) OR TITLE-ABS ( "electric stimulation therap*" ) OR TITLE-ABS ( neurostimulat* ) OR TITLE-ABS ( "Transcutaneous Electrical Stimulat*" ) OR TITLE-ABS ( "Transdermal Electrostimulat*" ) OR TITLE-ABS ( "Transcutaneous Electrical Nerve Stimulat*" ) OR TITLE-ABS ( "Transcutaneous Nerve Stimulat*" ) OR TITLE-ABS ( "Percutaneous Neuromodulation Therap*" ) OR TITLE-ABS ( "Percutaneous Electrical Neuromodulat*" ) OR TITLE-ABS ( "Percutaneous Electrical Neuromodulat*" ) OR TITLE-ABS ( "percutaneous electrical stimulat*" ) OR TITLE-ABS ( "transcutaneous electrical nerve stimulat*" ) OR TITLE-ABS ( "Transcutaneous neuromodulat*" ) ) )

2. Children concept: 5,563,354 hits

INDEXTERMS ( child ) OR INDEXTERMS ( pediatrics ) OR TITLE-ABS ( child ) OR TITLE-ABS ( child* ) OR TITLE-ABS ( schoolchild* ) OR TITLE-ABS ( schoolchild ) OR TITLE-ABS ( "school child" ) OR TITLE-ABS ( "school child*" ) OR TITLE-ABS ( kid ) OR TITLE-ABS ( kids ) OR TITLE-ABS ( toddler* ) OR TITLE-ABS ( adolescent ) OR TITLE-ABS ( adoles* ) OR TITLE-ABS ( teen* ) OR TITLE-ABS ( boy* ) OR TITLE-ABS ( girl* ) OR TITLE-ABS ( minors ) OR TITLE-ABS ( minors* ) OR TITLE-ABS ( underag* ) OR TITLE-ABS ( "under ag*" ) OR TITLE-ABS ( juvenil* ) OR TITLE-ABS ( youth* ) OR TITLE-ABS ( kindergar* ) OR TITLE-ABS ( puberty ) OR TITLE-ABS ( puber* ) OR TITLE-ABS ( pubescen* ) OR TITLE-ABS ( prepubescen* ) OR TITLE-ABS ( prepuberty* ) OR TITLE-ABS ( pediatric* ) OR TITLE-ABS ( paediatric* ) OR TITLE-ABS ( peadiatric* ) OR TITLE-ABS ( schools ) OR TITLE-ABS ( "nursery school*" ) OR TITLE-ABS ( preschool* ) OR TITLE-ABS ( "pre school*" ) OR TITLE-ABS ( "primary school*" ) OR TITLE-ABS ( "secondary school*" ) OR TITLE-ABS ( "elementary school*" ) OR TITLE-ABS ( "elementary school" ) OR TITLE-ABS ( "high school*" ) OR TITLE-ABS ( highschool* ) OR TITLE-ABS ( "school age" ) OR TITLE-ABS ( schoolage ) OR TITLE-ABS ( "school age*" ) OR TITLE-ABS ( schoolage* )

3. Lower Urinary Tract Dysfunction concept: 308,909 hits

TITLE-ABS ( "urinary tract*" ) OR TITLE-ABS ( "urinary symptom*" ) OR TITLE-ABS ( "urodynamic test*" ) OR INDEXTERMS ( "Lower Urinary Tract Symptoms" ) OR INDEXTERMS ( "urinary bladder, overactive" ) OR TITLE-ABS ( "Overactive Bladder" ) OR TITLE-ABS ( "Overactive Urinary Bladder" ) OR TITLE-ABS ( "Overactive Detrusor*" ) OR INDEXTERMS ( "urinary tract" ) OR INDEXTERMS ( "urinary incontinence" ) OR TITLE-ABS ( "urinary incontinence" ) OR INDEXTERMS ( "urinary bladder diseases" ) OR TITLE-ABS ( "bladder disease*" ) OR INDEXTERMS ( "urination disorders" ) OR TITLE-ABS ( "urination disorder*" ) OR INDEXTERMS ( urodynamics ) OR TITLE-ABS ( urodynamic* ) OR TITLE-ABS ( "urodynamic parameter*" ) OR TITLE-ABS ( "urge incontinence" ) OR TITLE-ABS ( "hyperactive bladder" ) OR TITLE-ABS ( enuresis ) OR TITLE-ABS ( "urinary urge incontinence" ) OR TITLE-ABS ( "urge syndrome" )

4. Combination #1 AND #2 AND #3: 141 hits
