# Supplementary material for: Systematic Review of Percutaneous and Transcutaneous Posterior Tibial Neurostimulation for Lower Urinary Tract Symptoms & Lower Urinary Tract Dysfunction in Children
Source: Neurourol Urodyn. 2026 Mar 15;45(4):774–93. doi: 10.1002/nau.70264 (PMC13054631; doi:10.1002/nau.70264)
Supplement: Supplementary file 2 — Supplemental Table 1. Full Mixed Methods Assessment Tool quality assessment ratings for included studies. [file NAU-45-774-s002.docx]

Supplemental Table 1. Full Mixed Methods Assessment Tool quality assessment ratings for included studies. LUTD = lower urinary tract dysfunction; RCT = randomized controlled trial.

| **Indication for PTNS/TTNS** | | **Study ID** | **Overall quality** | **All studies** | | | **1. Qualitative** | | | | | **2. Quantitative RCT** | | | | | **3. Quantitative non-randomized** | | | | | **4. Quantitative descriptive** | | | | | **5. Mixed Methods** | | | | |
| --- | --- | --- | --- | --- | --- | --- | --- | --- | --- | --- | --- | --- | --- | --- | --- | --- | --- | --- | --- | --- | --- | --- | --- | --- | --- | --- | --- | --- | --- | --- | --- |
|  | |  |  | **S1** | **S2** | | **1.1** | **1.2** | **1.3** | **1.4** | **1.5** | **2.1** | **2.2** | **2.3** | **2.4** | **2.5** | **3.1** | **3.2** | **3.3** | **3.4** | **3.5** | **4.1** | **4.2** | **4.3** | **4.4** | **4.5** | **5.1** | **5.2** | **5.3** | **5.4** | **5.5** |
| **Mixed neurogenic/**  **non-neurogenic LUTD** | | **Ansari et al, 2020** | 1 | Yes | Yes | |  |  |  |  |  |  |  |  |  |  |  |  |  |  |  | Can't tell | Can't tell | Yes | Can't tell | No |  |  |  |  |  |
|  |  | **Capitanucci et al, 2009** | 4 | Yes | Yes | |  |  |  |  |  |  |  |  |  |  |  |  |  |  |  | Yes | Yes | Yes | Can't tell | Yes |  |  |  |  |  |
|  |  | **Cardona-Grau et al, 2018** | 4 | Yes | Yes | |  |  |  |  |  |  |  |  |  |  |  |  |  |  |  | Yes | Yes | Yes | Can't tell | Yes |  |  |  |  |  |
|  |  | **DeGennaro et al, 2004** | 3 | Yes | Yes | |  |  |  |  |  |  |  |  |  |  |  |  |  |  |  | Can't tell | Yes | Yes | Can't tell | Yes |  |  |  |  |  |
|  |  | **Lecompte et al, 2015** | 4 | Yes | Yes | |  |  |  |  |  |  |  |  |  |  |  |  |  |  |  | Yes | Yes | Yes | Can't tell | Yes |  |  |  |  |  |
|  |  | **Roić et al, 2021** | 4 | Yes | Yes | |  |  |  |  |  |  |  |  |  |  |  |  |  |  |  | Yes | Yes | Yes | Can't tell | Yes |  |  |  |  |  |
|  |  | **Taverna et al, 2016** | 0 | Yes | Yes | |  |  |  |  |  |  |  |  |  |  |  |  |  |  |  | Can't tell | Can't tell | Can't tell | Can't tell | Can't tell |  |  |  |  |  |
|  | |  |  |  |  | |  |  |  |  |  |  |  |  |  |  |  |  |  |  |  |  |  |  |  |  |  |  |  |  |  |
| **Nocturnal enuresis** | | **AboulEla et al, 2014** | 0 | Yes | Yes | |  |  |  |  |  |  |  |  |  |  | Can't tell | Can't tell | Can't tell | No | Can't tell |  |  |  |  |  |  |  |  |  |  |
|  |  | **AlZamil et al, 2020** | 1 | Yes | Yes | |  |  |  |  |  |  |  |  |  |  |  |  |  |  |  | Can't tell | Can't tell | Yes | Can't tell | No |  |  |  |  |  |
|  |  | **Elshafey et al, 2015** | 2 | Yes | Yes | |  |  |  |  |  | Yes | Yes | Can't tell | No | Can't tell |  |  |  |  |  |  |  |  |  |  |  |  |  |  |  |
|  |  | **Ferroni et al, 2017** | 5 | Yes | Yes | |  |  |  |  |  |  |  |  |  |  |  |  |  |  |  | Yes | Yes | Yes | Yes | Yes |  |  |  |  |  |
|  |  | **Amar et al, 2020** | 2 | Yes | Yes | |  |  |  |  |  |  |  |  |  |  | Yes | Yes | Can't tell | Can't tell | Can't tell |  |  |  |  |  |  |  |  |  |  |
|  |  | **Perez-Martinez et al, 2020** | 0 | Yes | Yes | |  |  |  |  |  | Can't tell | Can't tell | Can't tell | Can't tell | Can't tell |  |  |  |  |  |  |  |  |  |  |  |  |  |  |  |
|  |  | **Raheem et al, 2013** | 4 | Yes | Yes | |  |  |  |  |  | Yes | Yes | Yes | Can't tell | Yes |  |  |  |  |  |  |  |  |  |  |  |  |  |  |  |
|  |  | **Vasudevan et al, 2018** | 1 | Yes | Yes | |  |  |  |  |  | Can't tell | Can't tell | Yes | Can't tell | Can't tell |  |  |  |  |  |  |  |  |  |  |  |  |  |  |  |
|  | |  |  |  |  | |  |  |  |  |  |  |  |  |  |  |  |  |  |  |  |  |  |  |  |  |  |  |  |  |  |
| **Non-neurogenic LUTD** | | **BarrosoJr et al, 2013** | 5 | Yes | Yes | |  |  |  |  |  |  |  |  |  |  | Yes | Yes | Yes | Yes | Yes |  |  |  |  |  |  |  |  |  |  |
|  |  | **Boudaoud et al, 2015** | 5 | Yes | Yes | |  |  |  |  |  | Yes | Yes | Yes | Yes | Yes |  |  |  |  |  |  |  |  |  |  |  |  |  |  |  |
|  |  | **DeWall et al, 2022** | 5 | Yes | Yes | |  |  |  |  |  |  |  |  |  |  |  |  |  |  |  |  |  |  |  |  | Yes | Yes | Yes | Yes | Yes |
|  |  | **Hoebeke et al, 2002** | 4 | Yes | Yes | |  |  |  |  |  |  |  |  |  |  |  |  |  |  |  | Can't tell | Yes | Yes | Yes | Yes |  |  |  |  |  |
|  |  | **Ibrahim et al, 2019** | 2 | Yes | Yes | |  |  |  |  |  |  |  |  |  |  |  |  |  |  |  | Can't tell | Can't tell | Yes | Can't tell | Yes |  |  |  |  |  |
|  |  | **Jafarov et al, 2021** | 3 | Yes | Yes | |  |  |  |  |  | Yes | Yes | Yes | Can't tell | Can't tell |  |  |  |  |  |  |  |  |  |  |  |  |  |  |  |
|  |  | **Mendes et al, 2016** | 5 | Yes | Yes | |  |  |  |  |  |  |  |  |  |  |  |  |  |  |  | Yes | Yes | Yes | Yes | Yes |  |  |  |  |  |
|  |  | **Patidar et al, 2015** | 2 | Yes | Yes | |  |  |  |  |  | Can't tell | Yes | Yes | Can't tell | Can't tell |  |  |  |  |  |  |  |  |  |  |  |  |  |  |  |
|  | **Key** | | | | |  |  |  |  |  |  |  |  |  |  |  |  |  |  |  |  |  |  |  |  |  |  |  |  |  |  |
|  | High quality (5/5 criteria met) | | | | |  |  |  |  |  |  |  |  |  |  |  |  |  |  |  |  |  |  |  |  |  |  |  |  |  |  |
|  | Moderate quality (3/5 or 4/5 criteria met) | | | | |  |  |  |  |  |  |  |  |  |  |  |  |  |  |  |  |  |  |  |  |  |  |  |  |  |  |
|  | Poor quality (1/5 or 2/5 criteria met) | | | | |  |  |  |  |  |  |  |  |  |  |  |  |  |  |  |  |  |  |  |  |  |  |  |  |  |  |
