# Supplementary material for: Systematic Review of Percutaneous and Transcutaneous Posterior Tibial Neurostimulation for Lower Urinary Tract Symptoms & Lower Urinary Tract Dysfunction in Children
Source: Neurourol Urodyn. 2026 Mar 15;45(4):774–93. doi: 10.1002/nau.70264 (PMC13054631; doi:10.1002/nau.70264)
Supplement: Supplementary file 3 — Supplemental Table 5. GRADE approach for certainty assessment of outcomes. [file NAU-45-774-s001.docx]

Supplemental Table 5. GRADE approach for certainty assessment of outcomes. LUTD = lower urinary tract dysfunction; LUTS = lower urinary tract symptoms; RCT = randomized controlled trial.

| Outcome information | | | | Certainty assessment | | | | |
| --- | --- | --- | --- | --- | --- | --- | --- | --- |
| Population | Outcome | Effect | Number of participants (study design)  Longest follow up | Risk of bias | Inconsistency | Indirectness | Imprecision | Certainty |
| Mixed neurogenic/non-neurogenic LUTD | **LUTS improvement** (Capitanucci et al., 2009; Cardona Grau et al., 2018; Lecompte et al., 2015; Roic et al., 2021; DeGennaro et al., 2004;, Ansari et al., 2020; Taverna et al., 2016) | Most studies showed >70% improvement in symptoms; variable degree of improvement for neurogenic LUTD | 182; 78 neurogenic  (7 quantitative descriptive studies)  3.5 years | Serious | Not serious | Not serious | Serious | Low |
| Nocturnal enuresis | **Frequency of wet nights** (Ferroni et al., 2017; Raheem et al., 2013; Elshafey et al., 2015; AlZamil et al., 2020; Vasudevan et al., 2018) | Most studies showed decreases of 1-2 nights over 1-2 weeks | 203  (3 RCTs, 1 quantitative non-randomized study, 1 quantitative descriptive studies)  4 months | Serious | Not Serious | Not serious | Serious | Low |
| Non-neurogenic LUTD | **Overactive bladder** (Barroso Jr. et al., 2013; Boudaoud et al., 2015; Ibrahim et al., 2019; Patidar et al., 2015) | About 60-70% of patients had at least some improvement; cure rates variable | 139  (2 RCTs, 1 quantitative non-randomized study, 1 quantitative descriptive study)  6 months | Serious | Serious | Not serious | Serious | Low |
| Non-neurogenic LUTD | **Dysfunctional voiding** (Hoebeke et al., 2002; Mendes et al., 2016) | About 50% had improved flow pattern; minimal to no improvements in continence | 51  (2 quantitative descriptive studies)  12 weeks | Serious | Not serious | Not serious | Serious | Low |
