# Supplementary material for: Systematic Review of Percutaneous and Transcutaneous Posterior Tibial Neurostimulation for Lower Urinary Tract Symptoms & Lower Urinary Tract Dysfunction in Children
Source: Neurourol Urodyn. 2026 Mar 15;45(4):774–93. doi: 10.1002/nau.70264 (PMC13054631; doi:10.1002/nau.70264)
Supplement: Supplementary file 4 — Supplemental Table 2. Full results of studies on tibial nerve stimulation for mixed neurogenic/non‐neurogenic LUTD (7 studies; 1 purely neurogenic). Supplemental Table 3. Full results of studies on tibial nerve stimulation for nocturnal enuresis (8 studies). Supplemental Table 4. Full results of studies on tibial nerve stimulation for non‐neurogenic LUTD (8 studies). [file NAU-45-774-s003.docx]

Supplemental Table 2. Full results of studies on tibial nerve stimulation for mixed neurogenic/non-neurogenic LUTD (7 studies; 1 purely neurogenic). 18F-FDG PET/CT = 18-fluorodeoxyglucose positron emission tomography/computed tomography; DVSS = dysfunctional voiding symptom score; NGB = neurogenic bladder; OAB = overactive bladder; PTNS = percutaneous tibial nerve stimulation; PVR = post-void residual; Qmax = maximum flow; TNS = tibial nerve stimulation; TTNS = transcutaneous tibial nerve stimulation; UDS = urodynamic studies; UR = urinary retention.

| Study & TNS treatment modality | Subjective Outcomes | Questionnaire Scores | Objective Outcomes |
| --- | --- | --- | --- |
| Ansari et al., 2020  PTNS | 54% (12/21) clinically improved  OAB:  58.3% (7/12) improved, of which:  43% (3/7) completely cured  43% (3/7) improved  14% (1/7) partially improved  33% (4/12) had no clinical benefit despite changes in brain physiology  Underactive bladder:  56% (5/9) improved, of which:  40% (2/5) completely cured  40% (2/5) improved  20% (1/5) partially improved  44% (4/5) had no clinical benefit despite changes in brain physiology |  | Brain activity on 18F-FDG PET/CT:  OAB: Decreased brain activity in “areas involved in the [sensation] of bladder filling” (mid-cingulate gyrus, hypothalamus, premotor cortex, lateral pons)  Underactive detrusor: avid uptake noted in “areas involved in sensorimotor learning and the initiation of voiding” (lateral cingulate gyrus, mid pons and periaqueductal grey). Unclear how this compared before or after treatment. |
| Cardona-Grau et al., 2018  TTNS | 79% (22/28) subjectively improved  75% (21/28) subjectively complied with treatment | Change in DVSS score:  OAB group: -5.5 (95% CI 3-8, p=0.004)  Not reported for NGB group. | 50% (14/28) had objective improvement based on “clinical parameters”  No significant changes in PVR any group, but greatest mean reduction in patients with incomplete emptying (-36 cc)  75% (21/28) completed >50% of treatments (measured by TENS unit) |
| Capitanucci et al., 2009  PTNS | Non-neurogenic: 78% improved  NGB: 14% improved  Underactive bladder: 82% improved  Underactive valve bladder: 52% improved  OAB: 42% (5/12) cured  DV: 86% (12/14) cured  Cure at 1 and 2 years:  71% of DV, 41% of OAB  Chronic stimulation needed to maintain results:  29% of DV, 50% of OAB (p not significant) |  | Normalization of uroflow voided volume:  57% of DV, 20% of OAB  Normalization of uroflow PVR:  57% of DV, 25% of OAB |
| DeGennaro et al., 2004  PTNS | OAB: 80% (8/10) improved  Incontinence associated with nighttime wetting: 44% (4/9) completely dry  UR: 71% (5/7) improved, 33% (1/3) achieved continence.  NGB: no significant improvement in symptoms. | Anxiety-depression test: Anxiety-depressive trait in 6/10 children and 7/10 parents.  Faces Pain Rating Scale (0-10, where 10 = most pain):  Patients: increased from minimum score 4 to 8 during sessions; stable across treatment course.  Parents: decreased from maximum score 5 to 2 during sessions; stable across treatment course.  Children's Hospital of Eastern Ontario Pain Scale (4-13, where 13 = most pain):  Patients: decreased from max scores 3-7 across multiple domains (crying, complaint, stiffness, legs, hands) to 0 at 30 minutes; lower starting scores with additional sessions.  Visual Analog Scale (1-10, where 10 = most pain):  Low mean level of pain, decreasing between maximum mean score 2.16 to minimum mean score 0.9 during sessions, stable across treatment course.  5.Questionario Italiano del Dolore (QUID):  Statistically significant decreases in scores across all components (sensitive pain, affective, mixed) from maximum mean score 1.3 to minimum mean score 0.10). | OAB:  DO disappeared in the 4/9 children who became dry.  Low cystometric bladder capacity normalized in 63% (5/8).  Mean cystometric bladder capacity increased from 192.8 +/- 75.8 to 236.5 +/- 87 ml (p=0.1).  UR:  -Significant PVR (>20% of cystometric bladder capacity) disappeared in 50% (3/6).  -Mean pressure at Qmax increased from 33.8 +/- 22.1 to 50.2 +/- 15.5 cm H2O (p=0.09).  -Mean Qmax increased from from 6.2 +/- 3.7 to 12.3 +/- 6.7 ml per second (p=0.05).  NGB:  -No significant improvements.  -Mean cystometric bladder capacity increased from 239 +/- 110 to 289 +/- 28 ml (p=0.2)  -Mean PVR decreased from 173 +/- 99 to 154 +/- 102 ml (p=0.3).  -Pressure at Qmax increased from 20 to 48 cm H2O in only 1 patient. |
| Lecompte et al., 2015  TTNS | 83% dry at 6 months All patients using anticholinergics stopped the medication. | Schurch urinary incontinence score: for those not dry, decrease from mean 1.6 to mean 0.5 at 2 months and mean 0.17 at 6 months.  0: completely dry; 1: wet once a day (mild); 2: wet for <50% of the time between CIC or spontaneous miction (moderate); and 3: wet for >50% of the time between CIC or spontaneous miction (severe) |  |
| Taverna et al., 2016  PTNS | 87.5% (7/8) had improvement in urgency and UI symptoms at 12 weeks (except for 1 with non-neurogenic OAB).  75% (3/4) of non-neurogenic group had disappearance of diurnal incontinence on 3rd week of treatment, of which 67% (2/3) had improvement in voided volume after 12 weeks.    All 3 patients with improvement in urodynamic tests post-treatment had resolution of nocturnal enuresis after 6 months and maintenance of clinical results at 3 years follow-up.  All patients (4/4) in spina bifida neurogenic OAB group had complete disappearance of diurnal incontinence after 12 weeks of treatment and maintenance of clinical results at 6-24 months of follow-up. |  | No changes in cystometric bladder capacity or detrusor overactivity on UDS  All 3 patients with improvement in urodynamic tests post-treatment had maintenance of urodynamic results at 3 years follow-up.  All patients (4/4) in spina bifida neurogenic OAB group had maintenance of urodynamic results at 6-24 months of follow-up |
| Roić et al., 2021  TTNS | 20% (8/39) had no symptom improvement.  30.8% (12/39) had a better sensation of bladder fullness.  15.4% (6/39) achieved urinary continence.  10.2% (4/39) voided with less straining. |  |  |

Supplemental Table 3. Full results of studies on tibial nerve stimulation for nocturnal enuresis (8 studies). DES = dysfunctional elimination syndrome; NLUTD = non-neurogenic lower urinary tract dysfunction; PedsQL = Pediatric Quality of Life Inventory; PIN-Q = pediatric incontinence questionnaire quality of life; PTENS = parasacral transcutaneous electrical nerve stimulation; PTNS = percutaneous tibial nerve stimulation; SD = standard deviation; TNS = tibial nerve stimulation; TTNS = transcutaneous tibial nerve stimulation;

| Study & TNS treatment modality | Subjective Outcomes | Questionnaire Scores | Objective Outcomes |
| --- | --- | --- | --- |
| Aboul Ela et al., 2014  PTNS | 60% (12/20) improved |  | 60% (12/20) had improvement of all urodynamic parameters with increase in bladder capacity and disappearance of detrusor instability. |
| AlZamil et al., 2020  TTNS | Average frequency of wet nights per week:  3.5±1.6 in high-frequency/low-amplitude group  1.4±1.1 in low-frequency/high-amplitude group  Low-frequency high-amplitude (LH) group: 20% (2/10) had full recovery (not defined).  1.7x more effective in low-frequency/high-amplitude group group compared to high-frequency low-amplitude group. Unclear how this was determined. |  | High-frequency/low-amplitude group had no significant changes in theta index on EEG activity.  Low-frequency/high-amplitude group group had 39% decrease in theta index. |
| Amar et al., 2020  PTNS | Frequency of nocturnal enuresis was improved in both PTNS and desmopressin groups (statistically significant). No significant differences between groups.  Relapse rate after 1 month of follow up (values not reported) was statistically significant in both groups and not significantly different between groups. |  |  |
| Elshafey et al., 2015  TTNS | 87.5% recovery rate (not well defined) with TTNS vs. 75% with bedwetting alarm (p=0.04).  Mean (SD) frequency of nocturnal enuresis decreased from 5.90 (0.41) to 1.20 (0.14) with TTNS (p=0.001), vs. 6.30 (0.23) to 3.40 (0.35) with bedwetting alarm (p=0.001). | 1.Mean (SD) KIDSCREEN-10 Index score (health-related quality of life) increased from 1.80 (0.04) to 3.90 (0.73) with TTNS (p=0.001), vs. 1.56 (0.09) to 2.80 (0.05) (p=0.001) with bedwetting alarm. | Mean (SD) maximum voided volume (mL) increased from 160 (15) to 192 (12) for TTNS (p=0.001), vs. 155 (14) to 176 (10) for bedwetting alarm (p=0.001). |
| Ferroni et al., 2017  TTNS | 72.7% (16/22) had at least 1 fewer wet night during the treatment period.  Mean (SD) total wet nights:  Entire cohort:  9 (4) pre-stimulation  6.8 (4.8) during stimulation (p<0.01 vs. pre)  7.2 (5.0) post-stimulation (p=0.02 vs. pre)  Responders: 7.9 (3.7) pre-stimulation  4.8 (3.5) during stimulation  5.1 (4.0) post-stimulation  Non-responders: 12.0 (NR) pre-stimulation | PedsQL questionnaire:  No significant differences between the periods before, during, or after stimulation for the entire cohort or responders alone.  Vancouver NLUTD/DES questionnaire:  No significant differences between the periods before, during, or after stimulation for the entire cohort.  Decreased for responders (mean 13.9 before, 11.6 during, and 10.5 after stimulation). | Mean age (range):  Responders: 12 (8-16) Nonresponders: 8.7 (7-10) (p<0.01) |
| Perez-Martinez et al., 2020  PTNS | Mean (SD) patient improvement (defined as "dry nights”):  80% (7.5%) for PTNS  3.7% (1.2%) for sham (p<0.001)  Mean (SD) dry nights 1 month after treatment:  92.5% (1.4%) for PTNS  10% (3.5%) for sham (p<0.001) |  |  |
| Raheem et al., 2013  PTNS | PTNS:  29% (4/14) full response  50% (7/14) partial response  21% (3/14) no response  Sham:  0% (0/14) full response  14% (2/14) partial response  86% (12/14) no response  Post-treatment comparison between groups: p=0.002.  Number of wet nights per week (mean +/- SD) decreased from 4.7 +/- 1.3 to 2.6 +/- 2.2 for PTNS (p=0.002), vs. 5.1 +/- 1.4 to 4.7 +/- 2.1 for sham (p=0.08); lower for PTNS post-treatment (p=0.041). |  | Volume at first desire to void (mL, mean +/- SD) increased from 148.46 +/- 25.89 to  177.71 +/- 35.48 for PTNS (p=0.002), vs. 153.50 +/- 21.65 to 154.14 +/- 20.71 for sham (p=0.59); higher for PTNS post-treatment (p=0.041).  Volume at strong desire to void (mL, mean +/- SD) increased from 260.43 +/- 84.18 to  283.64 +/- 72.03 for PTNS (p=0.01), vs. 271.79 +/- 75.43 to 271.6 +/- 72.8 for sham (p=0.94); higher for PTNS post-treatment (p=0.67).  Maximum cystometric capacity (mL, mean +/- SD) increased from 291.21 +/- 86.82 to  322.5 +/- 65.89 for PTNS (p=0.000), vs. 322.21 +/- 76.04 to 323.57 +/- 77.44 for sham (p=0.57); not significantly different between groups post-treatment (p=0.97).  Detrusor overactivity decreased from 50% (7/14) to 36% (5/14) for PTNS (p=0.44), vs. 43% (6/14) to 43% (6/14) with sham (p=0.44); not significantly different between groups post-treatment (p=0.7).  Maximum voided volume (mL, mean +/- SD) increased from 266.57 +/- 82 to 280.14 +/- 71.81 for PTNS (p=0.022), vs. 288.93 +/- 106.29 to 291.07 +/- 96.84 for sham (p=0.78); not significantly different between groups post-treatment (p=0.6). |
| Vasudevan et al., 2018  TTNS | Significantly fewer wet nights for parasacral TENS (p=0.003). Unclear if compared to baseline vs. other groups (such as PTNS).  TTNS group had mean (SD) decrease in wet nights -4.2% (5.9%), more than sham and PTENS groups but not statistically significant (p=0.128). | PIN-Q score: improved in all groups, but no significant differences between PTNS/PTENS/sham groups at months 1 (p=0.446), 2 (p=0.858), or 3 (p=0.737). |  |

Supplemental Table 4. Full results of studies on tibial nerve stimulation for non-neurogenic LUTD (8 studies). DVISS = Dysfunctional Voiding and Incontinence Symptom Score; DVSS = dysfunctional voiding symptom score; PIN-Q = pediatric incontinence questionnaire; PTENS = parasacral transcutaneous electrical nerve stimulation; PTNS = percutaneous tibial nerve stimulation; PVR = post-void residual; QOL = quality of life; TNS = tibial nerve stimulation; TTNS = transcutaneous tibial nerve stimulation; SD = standard deviation; UDS = urodynamic studies; UTI = urinary tract infection.

| Study & TNS treatment modality | Subjective outcomes | Questionnaire Scores | Objective Outcomes |
| --- | --- | --- | --- |
| Barroso Jr. et al., 2013  PTNS | Parent-reported full response: 9% for PTNS vs. 70% for PTENS (p=0.02)  Persistent urgency after treatment: 5 (23%) for PTNS vs. 4 (12%) for PTENS (p=0.294)  Persistent diurnal incontinence after treatment 9 (41%) for PTNS vs. 7 (20%) for PTENS (p=0.134).  Persistent enuresis after treatment: 14 (64%) for PTNS vs. 20 (59%) for PTENS (p=0.785). | Mean +/- SD DVSS decreased from 10.1 +/- 5.0 to 2.5 +/- 3.1 for PTNS, vs. 10.6 +/- 5.0 to 2.3 +/- 3.1 for PTENS (p=0.55) |  |
| Boudaoud et al., 2015  TTNS | Felt stimulation: TTNS 85%, sham 71%. | Mean (SD) urinary score (0 best, 13 worst):  TTNS 5.72 (2.14) to 6.18 (4.55)  Sham 5.66 (3.08) to 5.0 (3.87)  Results based on change in urinary score:  Very good: TTNS 45% (5/11), sham 66% (6/9)  Medium: TTNS 9% (1/11), sham 0%  Poor: TTNS 45% (5/11), sham 33% (3/9) | Mean voided volume during urgency episodes:  TTNS: 184 to 265 mL (p=0.002)  Sham: 184 to 181 (not significant)  Mean maximum cystomanometry volume:  TTNS: 215 to 274 mL (p=0.024)  Sham: not reported  Mean volume during first overactive detrusor contraction:  TTNS: 48 to 174 mL (p=0.001)  Sham: 61 to 80 mL (not significant)  Mean maximum detrusor pressure:  TTNS: 61 to 46 cm H2O (p=0.042)  Sham: 56 to 67 cm H2O (not significant) |
| DeWall et al., 2022  PTNS | 10% complete response (100% cure)  32% partial response (50-99% improvement)  58% no response (0-49% improvement)  Qualitative Data:  1. Decision to choose PTNS and expectations: ways to avoid surgery or medication or as a last resort  2. Time investment: travel time and having to leave school as downsides, preference for home treatment  3. Practical aspects of PTNS: nervousness before first session was common, absent to mild pain, distraction with books was helpful  4. Group setting / talking about LUTD: group setting provided reassurance to parents/patients, although varying degrees of interaction with others | Median child-reported PIN-Q score decreased (i.e. better QOL) from 25 to 19 (p=0.001)  Median parent-reported PIN-Q score decreased (i.e. better QOL) from 26 to 22 (p=0.001) | Median average voided volume (mL) increased from 114 to 139 (p=0.000)  Median maximum voided volume (mL) increased from 192 to 205 (p=0.025) |
| Hoebeke et al., 2002  PTNS | 84% (27/32) responded to treatment  Urgency: 28/31 before treatment, 11/31 after treatment (not significant).  Daytime incontinence: 23/31 before treatment, 19/31 after treatment (not significant).  17% became dry  52% had decreased incontinence  7% had no improvement  Disturbed voiding frequency (<4 or >8 voids per day): 19/31 before treatment, 3/31 after treatment (p<0.001)  No complications |  | Mean maximum bladder capacity increased from 185cc to 279cc (p<0.001).  Abnormal flow curve decreased from  21/31 to 12/31 patients (p=0.004) |
| Ibrahim et al., 2019  PTNS | 60% (12/20) requested to continue treatment (“success”)  60% (12/20) had improvement in daytime frequency  55% (11/20) had improvement in urgency  63% (10/16) had improvement in nocturnal enuresis  53% (8/15) had improvement in urge incontinence |  | Bladder capacity (mean ± SD) on UDS increased from 184.5±59.14 to 259.5±77.22 (p=0.001).  70% (14/20) had disappearance of involuntary detrusor contractions on UDS  57% (4/7) of patients with mild hypocompliance showed improvement in compliance.  20% (1/5) patients with moderate hypocompliance showed improvement in compliance. |
| Jafarov et al., 2021  TTNS | 2 weeks post-treatment:  Cured: TTNS 50% (10/20), sham 20% (2/10)  Improved: TTNS 20% (4/20), sham 40% (4/10)  No change: TTNS 30% (6/20), sham 40% (4/10)  2 years post-treatment:  Cured: TTNS 50% (10/20), sham 20% (2/10)  Improved: TTNS 20% (4/20), sham 50% (5/10)  No change: TTNS 30% (6/20), sham 30% (3/10)  Daytime episodes of urgency (mean ± SD):  TTNS:  BT: 4.9 ± 4.9 AT: 5.20 ± 6.66 LT: 3.4 ± 3.80 No significant differences  Sham:  BT: 5.90 ± 6.85 AT: 5.20 ± 6.66 LT: 3.4 ± 3.80 No significant differences  Daytime episodes of incontinence (mean ± SD):  TTNS:  BT: 1.50 ± 2.17 AT: 0.90 ± 1.59 LT: 0.40 ± 0.52 No significant differences  Sham:  BT: 1.50 ± 2.17 AT: 0.90 ± 1.59 LT: 0.40 ± 0.52 No significant differences  Nocturnal episodes of incontinence (mean ± SD):  TTNS:  BT: 3.00 ± 3.24 AT: 2.15 ± 3.01 LT: 1.35 ± 2.25 No significant differences  Sham:  BT: 1.80 ± 2.39 AT: 0.60 ± 0.52 LT: 0.40 ¬± 0.70 No significant differences | DVISS score (mean ± SD):  TTNS:  Before treatment (BT): 15.65 ± 6.73 After treatment (AT): 7.25 ± 6.55 Long term after treatment (LT): 6.20 ± 5.04 Significant differences between BT and AT, BT and LT, but not AT & LT.  Sham:  BT: 17.30 ± 8.52 AT: 8.10 ± 5.76 LT: 5.90 ± 5.44  Significant differences between BT and AT, BT and LT, but not AT & LT.  DVISS QOL score (mean ± SD):  TTNS:  BT: 2.30 ± 0.82 AT: 1.20 ± 1.03 LT: 0.70 ± 0.67 Significant differences between BT and AT, BT and LT, but not AT & LT.  Sham:  BT: 2.30 ± 0.82 AT: 1.20 ± 1.03 LT: 0.70 ± 0.67 Significant differences between BT and AT, BT and LT, but not AT and LT.  DVISS nighttime score (mean ± SD):  TTNS:  BT: 3.80 ± 3.49 AT: 3.00 ± 2.58 LT: 1.20 ± 2.09 No significant differences.  Sham:  BT: 3.80 ± 3.49 AT: 3.00 ± 2.58 LT: 1.20 ± 2.09 No significant differences  DVISS daytime score (mean ± SD):  TTNS:  BT: 13.50 ± 4.70 AT: 5.10 ± 3.57 LT: 4.70 ± 3.56 Significant differences between BT and AT, BT and LT, but not AT and LT.  Sham:  BT: 13.50 ± 5.32 AT: 5.10 ± 3.57 LT: 4.70 ± 3.56 Significant differences between BT and AT, BT and LT, but not AT and LT. | No difference in urinary NGF, TGFβ-1, and TIMP-2 levels before or after treatment for TTNS or sham. |
| Mendes et al., 2016  PTNS | 33% had improved continence (p=1)  50% had improvement in UTIs (p=0.1)  50% had less abdominal straining (p=0.2) |  | Mean +/- SD flow time (seconds) decreased from 59 +/- 34 (range 20-140) to 39 +/- 15 seconds (range 9-54) (p=0.05).  Mean +/- SD PVR decreased from 146.6 +/- 176.1 mL (range 0-570) to 39.6 +/- 49.9 mL (range 0-150) (p=0.01).  Mean voided volume (mL) was 246.8 before PTNS and 284.6 after (p=0.2).  63% of patients had improved flow pattern (p=0.0001)  71% of patients had improved PVR (p=0.0002). |
| Patidar et al., 2015  TTNS | 67% (14/21) cure for TTNS vs. 0% (0/16) for sham  24% significantly improved for TTNS vs. 6% for sham  9% partial response for TTNS vs. 19% for sham  0% no response for TTNS vs. 75% for sham  Severity of incontinence before and after treatment:  TTNS:  None: 0% to 71% (15/21)  Mild: 33.33% (7/21) to 24% (5/21)  Moderate: 38% (8/21) to 5% (1/21)  Severe: 28.5% (6/21) to 0%  Sham:  None: 0% to 13% (2/16)  Mild: 31% (5/16) to 31%  Moderate: 43.75%% (7/16) to 38%  Severe: 25% (4/16) to 19% |  | Mean average voided volume (mL): increased from 68 to 89 for TTNS (p=0.001), vs. 74 to 79.5 for sham (p=0.088).  Mean maximum voided volume (mL) increased from 116 to 190 for TTNS (p=0.01), vs. 110 to 145.5 for sham (p=0.072).  Mean number of voids decreased from 11 to 7 for TTNS (p=0.001) vs. 10 to 10 for sham (p=0.325). |
